# “ChatGPT says no”: agency, trust, and blame in Twitter discourses after the launch of ChatGPT

**DOI:** 10.1007/s43681-023-00414-1

**Published:** 2024-01-23

**Authors:** Dan Heaton, Elena Nichele, Jeremie Clos, Joel E. Fischer

**Affiliations:** 1 School of Computer Science, University of Nottingham, Nottingham, Nottinghamshire UK; 2 Department of Management, College of Arts Social Sciences and Humanities, University of Lincoln, Lincoln, UK

**Keywords:** Large language model, Decision-making algorithm, Social actor representation, Corpus linguistics, Critical discourse analysis, Social media

## Abstract

ChatGPT, a chatbot using the GPT-n series large language model, has surged in popularity by providing conversation, assistance, and entertainment. This has raised questions about its agency and resulting implications on trust and blame, particularly when concerning its portrayal on social media platforms like Twitter. Understanding trust and blame is crucial for gauging public perception, reliance on, and adoption of AI-driven tools like ChatGPT. To explore ChatGPT’s perceived status as an algorithmic social actor and uncover implications for trust and blame through agency and transitivity, we examined 88,058 tweets about ChatGPT, published in a ‘hype period’ between November 2022 and March 2023, using Corpus Linguistics and Critical Discourse Analysis, underpinned by Social Actor Representation. Notably, ChatGPT was presented in tweets as a social actor on 87% of occasions, using personalisation and agency metaphor to emphasise its role in content creation, information dissemination, and influence. However, a dynamic presentation, oscillating between a creative social actor and an information source, reflected users’ uncertainty regarding its capabilities and, thus, blame attribution occurred. On 13% of occasions, ChatGPT was presented passively through backgrounding and exclusion. Here, the emphasis on ChatGPT’s role in informing and influencing underscores interactors’ reliance on it for information, bearing implications for information dissemination and trust in AI-generated content. Therefore, this study contributes to understanding the perceived social agency of decision-making algorithms and their implications on trust and blame, valuable to AI developers and policymakers and relevant in comprehending and dealing with power dynamics in today’s age of AI.

## Introduction

The academic debate surrounding the agency of automated decision-making algorithms is ongoing [1–3]. While these algorithms can reduce margin of error in their intended use cases [4–7], their social agency and human-like attributes emerge when they assume decision-making roles [8, 9]. When such algorithms deviate from expected performance, there can be severe consequences, risking biased outcomes, undermining trust and increasing the potential for blame. Trust in decision-making algorithms hinges on perceptions of agency and responsibility, with users favoring autonomously operating algorithms that consistently yield reliable outcomes [10, 11], while distrust arises when algorithms seem influenced by external factors like human biases [10]. As well as this, blame, shaped by moral judgments and cognitive biases, presents complexity due to the agency agency attributed to decision-making algorithms [12–14], resulting in a ‘responsibility gap’ and challenges in attributing blame for negative outcomes [15–17], particularly when algorithms perpetuate biases or generate negative results amid algorithmic opacity, such as the UK A Level algorithm in 2020 [18]. This indicates that implications for trust and blame may impact algorithmic adoption [10, 11]. Therefore, investigating algorithmic agency is crucial and can be particularly urgent if the algorithm itself backgrounds those who are responsible for its development and deployment [19–22], as in the case of the chatbot ChatGPT.

ChatGPT constitutes one example of a contemporary decision-making algorithm—an artificial intelligence chatbot, which was introduced by OpenAI in November 2022. ChatGPT leverages deep learning models and natural language processing techniques to engage in human-readable text-based conversations [23, 24]. Its versatility extends beyond casual conversation, as it has proven itself capable of providing assistance and entertainment [25–27]. With its remarkable ability to comprehend and respond to a diverse range of queries and prompts, ChatGPT has gained widespread recognition, boasting over 100 million monthly users [28–30]. This study focuses on a potential ‘hype period’ [31] of early popularity of ChatGPT—between its launch in November 2022 and the announcement of GPT-4 in March 2023.

A small number of studies have specifically explored ChatGPT’s agency. For instance, research into society’s perception of ChatGPT’s human-like traits [32–35] viewed the algorithm behind it as an author, interactor, and influencer. Other studies found conflicting narratives, with ChatGPT depicted as creative and beneficial, yet also as incompetent and polluting human culture [36]. Others emphasised the social agency conflicts affecting the adoption of an anthropomorphic perspective and treating AI as a social actor [37].

As mentioned, upon its launch, ChatGPT gained significant public attention, with social media platforms, especially Twitter, serving as a sounding board for user reaction to its deployment [38, 39]. While efforts have focused on gathering public perspectives on ChatGPT [28–30], a noticeable research gap pertains to the examination of views expressed on Twitter. This social media platform contains vast, diverse opinions on current events [40, 41], and hence, it can be a valuable source of data for understanding public reactions. Despite the initial studies investigating the perceived social agency of ChatGPT, a research gap remains as to its presentation on Twitter specifically.

Addressing this research gap can offer comprehensive and in-depth insights into the broader public’s reactions to ChatGPT. A small number of studies have applied NLP-based approaches to analyze Twitter reactions to ChatGPT [38, 39, 42, 43], although these studies have analysed general topic and sentiment trends only. Even though helpful in the identification of themes and ideas, the presentation of ChatGPT’s perceived agency is yet to be investigated.

To delve into the intricate relationship between grammatical agency and social agency, we integrated Corpus Linguistics (CL) and Critical Discourse Analysis (CDA) [44], underpinned by Social Actor Representation (SAR) [45]. Grammatical agency, often manifested through transitivity, can help distinguish whether an entity is portrayed as the performer of actions or the passive recipient of them [46]. For instance, studies have used NLP-based results to guide their investigation into the agency and social actor status of decision-making algorithms [18]. By scrutinising the discourse surrounding ChatGPT and decision-making algorithms, we can illuminate perceived power dynamics and identify social actors that emerge within these intricate discussions [45, 47], illuminating perspectives on trust and blame and the barriers to decision-making algorithm adoption.

In summary, this paper employs CL and CDA, underpinned by SAR, to analyze Twitter discourses surrounding ChatGPT, focusing on the implications of trust and blame through agency and transitivity. The contribution aims to answer the research question: in what ways has ChatGPT been portrayed as a social actor in Twitter discourses over an early period of its popularity, and what are the implications for trust and blame?

## Related work

### Agency and social actors

In this section, we delve into the intricate relationship between grammatical and social agency, examining how linguistic elements convey agency and the implications of decision-making algorithms’ social agency, particularly in the context of social media. It addresses the debates surrounding algorithmic autonomy, accountability, and blame attribution, highlighting the complexities in assigning responsibility due to algorithmic opacity. This section also discusses the crucial role of language in shaping the performative agency of AI-based algorithms and the potential consequences of presenting algorithms as social actors.

#### Grammatical and social agency

Defining agency involves recognising a clear, i.e., well defined or distinct, sense of control, and its influence on human consciousness, i.e., state of awareness and perception, encompassing the responsibility associated with an entity’s operations and their consequences [48, 49]. Agency, denoting the capacity for intentional action and influence, is central to human and group decision-making as it empowers individuals and collectives to make deliberate choices, shape their environments, and take responsibility for the outcomes of their actions. [50, 51]. It involves cognitive, behavioural, and motivational processes, shaped by social and cultural contexts [52, 53]. Agency is not static but dynamic, a negotiation between individuals and their environment—an example of this being the way people adapt their decision-making processes when facing new challenges or changing circumstances[54].

Social agency can be studied by examining grammatical agency, i.e., how linguistic elements convey agency. In this sense, grammatical agency defines an agent as an entity with internal energy sources that exert force to perform actions within the text [46]. Within texts, linguistic agency is communicated by emphasising, manipulating, or concealing such an action [44]. A way to explore agency in this regard is through transitivity analysis, which involves examining the use of active and passive voice, as well as nominalisation, where verbs are converted into nouns. While other analytical procedures—such as pragmatics—may offer valuable insights into contextual meaning and language use, our study aimed to specifically dissect the grammatical nuances that attribute agency to AI. Analyzing these linguistic structures provides a unique lens to unravel the perceptions and representations of AI-driven tools within social interactions on Twitter.

Overall, linguistic choices reflect the language user’s attitudes, ideologies, and perceived agency [47, 55]. For example, research has shown that passive constructions tend to diminish the agency of the subject, particularly when the subject is absent from the clause, thus shifting implied responsibility onto entities present and foregrounded in the construction [47, 56]. Therefore, this dimension of agency relates to decision-making power. Alternatively, agency can also be conveyed through lexical choices. For instance, agency metaphors associate certain trajectories with animacy and inanimacy, reflecting the capacity for decision-making and the potential to finalise decisions [57, 58]. This is particularly pertinent to this context as ‘artificial intelligence’ itself is metaphorical.

#### Agency, social action, blame, and decision-making algorithms

Decision-making algorithms, such as large language models like GPT-n series and Google’s LaMDA, have gained increased attention, especially with regard to their perceived social agency and their role in addressing societal issues [1, 4]. In this context, a debate arises regarding the algorithms’ autonomy and agency [11, 59–61]. Some argue for algorithmic autonomy, whereby they can learn and adapt to the context [62]. However, it is a spectrum, including potential benefits (e.g., efficiency) and risks (e.g., biases). Contrarily, others assert that algorithms are not autonomous but influenced by human design choices, which raises ethical concerns [11, 63, 64]. While the legal aspects of these algorithms have been extensively studied [65–67], their social agency remains relatively unexplored, especially with regards to their interplay between algorithms and agency [60], whose importance in public life continues to grow.

Several studies have documented the tendency to treat AI as a distinct, i.e., overt and unique, social actor [68, 69]. For instance, [60] identified key issues regarding agency, autonomy and human respect in algorithmic decision-making. Despite research in areas such as journalism studies, where systems mainly work to inform [70–72], other studies highlight how algorithms may establish unmanageable rules, lack rationale for decisions, limit appeals, disregard interpersonal boundaries, and enable decision-makers to evade accountability.

Moreover, other studies found that automating decision-making can be dehumanising, especially within the healthcare sector and autonomous vehicles, yet algorithms can assist in stressful decisions [61, 73], possibly depending on the type of interaction happening between them and the humans [74]. Other existing literature suggests that, while concerns about agency are complex, algorithmic power implies a form of agency [75]. Following this, research has questioned whether algorithms should become more human-like or humans more akin to algorithms [8, 9, 76].

All these debates stem from the impact that decision-making algorithms can have on social agency, potentially limiting human accountability. In fact, opacity in machine learning algorithms blurs agency and transparency, which may perpetuate bias [77] by not highlighting data quality issues and potential model biases. Although transparency alone may not be enough to make complex data and models fully understandable [78], research has highlighted a lack of transparency in algorithms in areas like finance consequently erodes public agency [79]. Solutions proposed to ensure accountability include transparency and involving affected communities in system design [80, 81], since the level of data detail also affects fairness, hence agency.

Research by Heaton et al. suggests that the concepts of agency, accountability, and responsibility have a significant impact on trust and blame in relation to decision-making algorithms [59]. Their literature review highlights the need for a more nuanced understanding of the impact of these concepts on trust and blame and emphasises the importance of responsible and ethical governance of decision-making algorithms.

Humans sometimes resist using decision-making algorithms, influenced by factors at societal, algorithmic, and individual levels, including anthropomorphism, complexity, accuracy, and learning capabilities [82–85]. The emphasis on the use of machine learning and ‘black box’ techniques, meaning systems that produce results without clear or easily understandable explanations, to automate decisions creates a need for transparency in algorithm design and implementation [86]. Even though individuals behind these systems are rarely working in isolation (they are usually interchangeable parts contributing to a larger project [87, 88]), this collective effort does not reveal the inner workings of the system itself, necessitating the requirement for explainability and allowing light to be shed on decision-making processes [89]. Furthermore, morally sensitive decisions may be delegated to machines by humans, shifting responsibility, accountability, and blame from human decision-makers to AI-operated systems [18, 20].

To examine algorithms’ recurrent generation of bias and suboptimal decisions, understanding the pivotal role of language in the performative agency of AI-based algorithms is essential. This is because language, within real-world databases utilised for AI solutions, both represents and mirrors the diverse dimensions of social agency [90–92]. These algorithms carry out their tasks by converting language into categories, thereby establishing legitimacy and authority [75, 93]. In addition to merely using language, the developers of decision-making algorithms have created entities that closely replicate real-world interactions [94, 95]. Therefore, recognising the relationship between language and the performance of AI-based algorithms is crucial for addressing the recurrent issues of bias and suboptimal decisions.

Trust in decision-making algorithms depends on users’ perceptions of agency and responsibility. Studies indicate that users are more inclined to trust algorithms that operate autonomously and consistently produce reliable outcomes [10, 11]. In contrast, when algorithms are seen as influenced by external factors, such as human biases, their trustworthiness may be questioned [10]. Additionally, the fulfillment of responsibilities and accountability by actors involved in developing and deploying decision-making algorithms significantly impacts trust. When developers and operators meet their obligations and are held accountable for their actions, users tend to have greater trust in the overall integrity and reliability of the algorithms. Conversely, the failure to fulfill these responsibilities can result in blame assignment and reduced trust in the algorithms [10].

Blame, characterised by the assignment of responsibility with negative connotations and shaped by moral judgments and cognitive biases, plays a complex role in decision-making algorithms [12–14]. This complexity arises, because the high agency in algorithms can create a ‘responsibility gap’, necessitating clarity in attributing blame for negative outcomes [15–17]. Consequently, algorithms can have blame attributed to them, particularly when they perpetuate biases or produce negative outcomes, despite the complexities of assigning responsibility due to algorithmic opacity [18, 77, 96].

Recently, there is an emergence in investigating agency in decision-making algorithms on social media, likely due to the increasing use of AI and decision-making algorithms in various aspects of people’s lives. For example, Twitter users frequently attributed blame to an algorithm used to calculate A Level results in the UK in 2020 by presenting it as a standalone social actor [18]. Users often used active agency expressions, such as metaphors like ‘that algorithm is going to screw you’ and personalisation of the algorithm’s actions. This research not only sheds light on the public’s response to algorithmic decision-making but also holds broader implications for understanding future reactions to similar events, such as the launch of generative AI like ChatGPT.

In light of these complexities, it is worth questioning whether algorithms can possess human-like agency and exploring the implications of such a presentation on this topic.

### Background to ChatGPT

#### Timeline of ChatGPT

ChatGPT—short for Chat Generative Pre-trained Transformer—is an AI-powered conversational agent developed by OpenAI. It represents a groundbreaking advancement in mainstream technology, underpinned by deep learning models and natural language processing techniques [24]. This technology harnesses an extensive corpus of diverse textual data to engage in human-like conversations [23]. At its core, ChatGPT is empowered by a transformer-based language model, fine-tuned through reinforcement learning, enabling it to generate contextually relevant responses [97, 98]. Users interact with ChatGPT through a user-friendly interface, experiencing real-time, simulated natural conversations that span assistance, entertainment, and creative collaboration, marking an advancement in AI-driven conversational systems.

**Table 1:** Number of monthly users of ChatGPT [99]

Month	Number of visits	Change over previous month	Change over previous month (%)
November 2022	152.7 million	–	–
December 2022	266 million	113.3 million	74.2%
January 2023	616 million	350 million	131.58%
February 2023	1 billion	384 million	62.34%
March 2023	1.6 billion	600 million	60%

In November 2022, ChatGPT was **launched**, building upon OpenAI’s GPT-3 model, designed to engage users in conversations, challenge assumptions, and respond coherently [38, 100, 101]. Rapidly gaining popularity, ChatGPT surpassed 100 million monthly users in January 2023 (as shown in Table 1), attracting attention for its ability to pass graduate-level exams [28, 102]. However, accessibility issues and outages frustrated users [103].

As **popularity** of ChatGPT began to surge, OpenAI introduced the AI Text Classifier in January 2023 to tackle academic dishonesty [104, 105]. On February 1 2023, further **developments** began to arise ChatGPT Plus, a subscription plan, was launched to enhance user experience [106]. On March 1 2023, an API enabled seamless integration of ChatGPT into various applications, priced at $0.002 per 1,000 tokens [107]. Moreover, in March 2023, OpenAI unveiled GPT-4, an AI language model, and integrated it into various products, including ChatGPT, making it a multimodal system [108, 109].

#### Impact of ChatGPT

The social impact of ChatGPT has been profound [28–30]. It has stirred fascination and concern, fueling discussions about its benefits and drawbacks [30, 110]. Its applications span content generation, code debugging, data organisation, and therapy, impacting various industries [38, 111]. Yet, it has the potential to generate misinformation and displace jobs [112, 113]. Some ethical concerns arise from biases, privacy issues, and the risk of malicious use [114], much like other contemporary decision-making algorithms such as the NHS COVID-19 contact-tracing app [2, 115, 116]. Other non-exhaustive concerns include about its ability to generate “artificial hallucinations”, using copyrighted data, the "humans" behind the training, their working conditions. Therefore, research indicates the need for human oversight to manage biases and errors [117–119].

In turn, ChatGPT’s impact on other landscapes has continued to grow. For example, the political nature of ChatGPT has come under scrutiny, revealing its biases [120, 121]. Similarly, ChatGPT has also exacerbated challenges within educational contexts, such as academic dishonesty and plagiarism [122–124]. Nevertheless, ChatGPT’s full societal impact is still evolving, presenting both opportunities and challenges, necessitating ongoing research and ethical considerations [28–30].

In particular, a growing number of studies examine ChatGPT on Twitter. Notably, NLP-based tools have been used to explore public sentiments, particularly among early adopters. Haque et al. found generally positive sentiments and discussions on various topics, such as capabilities, limitations, industry impact, and ethics [42]. Taecharungroj identified three general topics (news, technology, and reactions) and five functional domains (creative writing, essay writing, prompt writing, code writing, and answering questions) [38]. Korkmaz et al. also revealed both positive and negative user experiences [39], while Leiter et al. identified major topics, like science and technology, learning and education, news and social concern, diaries and daily life, business, and entrepreneurs [43]. Finally, Heaton et al. analysed 88,058 tweets about ChatGPT from November 2022 to March 2023, with topic modeling results revealing diverse subjects, including text generation, chatbot development, data importance, API usage, comparisons with other companies, and cryptocurrency discussions [125]. Sentiment analysis showed predominantly positive sentiments, although they fluctuated over time, with a decline possibly linked to the ChatGPT Plus launch and algorithmic limitations. Emotion detection identified fluctuating patterns of ‘trust’ and ‘fear,’ with ‘trust’ declining alongside the ChatGPT Plus release, suggesting concerns about biases and misinformation.

However, there are limitations to these Twitter studies. The methodological limitations of using NLP-based tools in these studies caused difficulties in interpreting outputs and discrepancies between automated topic and emotion labelling and human review, underscoring concerns regarding accuracy. Solely relying on automated categorisation might neglect nuanced language aspects and result in accuracy gaps. Additionally, there is still limited research into the presentation of ChatGPT on Twitter with regard to its social and grammatical agency. With a focus on considering contextual factors and ChatGPT’s presentation as a potential social actor, this work intends to address these constraints and enhance comprehensiveness in exploring the Twitter discussion around ChatGPT by integrating NLP tools with additional approaches, like CL and CDA.

#### Agency, social action, and ChatGPT

To date, there has been a small number of studies that have specifically investigated the agency and social actor status of ChatGPT. In their study, Bran et al. found that four analysed news sources presented conflicting narratives about ChatGPT’s competence as a social actor, with some depicting it as creative and healing, offering valuable novelty, companionship the need for social acceptance [36]. Conversely, some sources portrayed AI as incompetent, polluting human culture, and replacing human skills and knowledge with stochastic, illusionary competence or even imposture. This dual representation leads to a contested social agency, vacillating between creative actors and essentially unthinking tools.

Additionally, Shijie et al. underscored the importance of evaluating the credibility of AI-generated content by considering AI’s role as a content generator and technological medium [37]. They highlighted the need to incorporate AI’s explainability and generative capabilities through third-party interface, while also keeping a significant focus on viewing AI through an anthropomorphic lens, treating it as a social actor to better assess its credibility.

Gutierrez stated that AI systems, such as ChatGPT, function as social intermediaries within a network of actors and associations, where they play a role in generating outputs and intentions [35]. In contrast to other findings, while AI lacks moral agency, its interactions have consequences, potentially including bias, accountability issues, transparency concerns, and privacy implications.

The tendency to treat ChatGPT as a social actor, in the same manner as other examples of AI, has been explored in an educational context by Dai et al. [126]. Through discussing the potential benefits and challenges of ChatGPT and generative AI in higher education, including its potential to enhance student learning, propel student engagement, impact academic integrity, and alter the role of educators, they found that ChatGPT has the potential to be a student-driven innovation. More of a focus was put on ChatGPT empowering students’ epistemic agency, but it is clear that careful consideration must be given to its implementation and use.

Research has also been undertaken into the perception of ChatGPT’s human-like traits in society by Al Lily et al., who analysed insights from 452 individuals worldwide, leading to the identification of two distinct categories of traits [32]. The first category revolved around social traits, where ChatGPT assumes the roles of an ‘author’ mirroring human phrasing and paraphrasing practices, and an ‘interactor’, emulating human collaboration and emotional engagement. The second category revolved around political traits, with ChatGPT adopting the roles of an ‘agent’, replicating human cognition and identity, and an ‘influencer’, simulating human diplomacy and consultation. Interestingly, ChatGPT itself acknowledged the possession of these human-like traits, reinforcing its role in human society as a ’semi-human’ actor that transcends its machine-based origins and technical essence.

Despite these studies, there is yet to be substantial research that focuses on how ChatGPT is presented on Twitter with regard to its perceived social agency, as this contribution intends to.

## Methods

### Data

Twitter was chosen as the platform for analysis due to its significant user base and the conversational nature of its content. However, it is important to note that this choice may not encapsulate the diverse landscape of social media platforms worldwide, and its findings might not universally represent all global demographics. Although data were global, location data were not captured and, thus, no geographical comparisons are able to be made. This study utilised the same dataset as Heaton et al. [125], with data extraction carried out using the Tweepy module in Python [127]. To capture discussions about ChatGPT’s operation and any tweets mentioning OpenAI, the search terms used to collect data were as follows: ‘chatgpt algorithm’, ‘chat gpt algorithm’, ‘chatgpt llm’, ‘chat gpt llm’, ‘chatgpt ‘large language model’, ‘chat gpt ‘large language model’, ‘chatgpt model’, ‘chat gpt model’, ‘chat gpt @openai’ and ‘chatgpt @openai’. A search for "ChatGPT" alone yielded an unmanageable number of results (over 3 million for 1 day alone). The dataset comprised 88,058 tweets collected from November 30 2022 (ChatGPT’s release) to March 6 2023 (a week before GPT-4’s launch to avoid confusion). While the data were global, we focused on tweets in English only.

Data collection involved the use of the Twitter for Academic Purposes API, chosen for its rich real-time data availability [128] and its suitability for pre-processing and exploratory analysis [129, 130]. Data pseudonymisation was implemented, with each tweet assigned a unique reference number. We systematically removed stopwords, lengthy and brief URLs, and ‘RT’ (retweet) indicators. Twitter handles within tweets were redacted for anonymity using gensim. While tweets are public by default, they are not necessarily published with the intent to be used for research purposes, and securing explicit user consent is often infeasible [131]. To align with best practices in social media research, we anonymised tweets during data cleaning and shared only concise verbatim excerpts. This project design was approved by our university department’s ethics committee.

### Corpus linguistics

We chose Corpus Linguistics (CL) for its ability to analyze large datasets efficiently and uncover language patterns by automatically ‘tagging’ words in texts with their corresponding word class [132]. CL offers various language-focused perspectives, including diachronic comparisons focusing on lexical usage [133]. Its effectiveness in revealing language patterns in substantial datasets [134–136] makes it a common choice for social media analysis [137–139]. CL also facilitates the comparison of multiple corpora, making it suitable for analysing data from different time periods, such as in this study. As the analysis focused solely on agency (grammatical and social), we used only corpus linguistics software that automatically assigns word class tags to text. Thus, this was the sole computational technique used.

In this analysis, we utilised CL-computerised tools to examine collocation, the co-occurrence of words within a defined span [132]. Rather than relying solely on frequency, it is possible to use statistical significance measures like LogDice and Log Likelihood to identify lexical and grammatical associations and themes [140]. Concordances, showing the proximity of related words, helped identify collocations [141].

The CL software used for this analysis was The Sketch Engine [142], chosen for its practicality and availability to academics. It also provided a series of reference corpora for comparison.

The analysis consisted of several stages. Concordance lines with ‘ChatGPT’ as a potential social actor were examined to initiate collocation analysis. We explored active and passive constructions. To identify active constructions, we established the collocation criteria of ‘ChatGPT’ and a single verb to the right (R1). To detect passive constructions, we employed the search criteria ‘by ChatGPT’. However, some passive presentations, exemplified by phrases like ‘ChatGPT was x-ed by...’ and ‘ChatGPT has been x-ed,’ were identified by including verbs to the right (R1). As a result, these passive structures were distinguished from active ones and re-classified accordingly.

LogDice was chosen as the statistical measure of collocational strength as it not only measures the statistical significance of a collocation, but it also factors in the size of the subcorpus, making comparisons between subcorpora of different sizes easier. To take advantage of this capacity, we split the corpus into three subcorpora that reflected the key moments in the evolution of ChatGPT, as illustrated earlier in the Related Work section, in chronological order. We did this to facilitate better comparisons in the timeline and answer our research question about how ChatGPT’s presentation differed as time progressed. These periods were:

- Period 1: Launch (November–December 2022)
- Period 2: Popularity (January 2023)
- Period 3: Developments (February and March 2023).

The strongest collocates for each period were reported based on LogDice scores, with a minimum threshold of three occurrences to determine significance. The top ten words with the strongest collocations from each time period were analysed using CDA, which will be explored methodologically next.

### Critical discourse analysis

Subsequently, we employed Critical Discourse Analysis (CDA). CDA, as an interpretative qualitative text analysis method, draws on relevant theoretical frameworks [143–145]. It aids in discerning implied meanings within the textual context [146–148]. This approach aligns with our data-driven strategy for addressing research questions regarding the app’s presentation and its impact on British society, a method well demonstrated in numerous studies involving Twitter discourses [149–151], showing that a specific focus on agency fosters the identification of blame and responsibility.

By combining CDA with the results from the Sketch Engine CL-analysis tool [142], we were able to delve into the agency and social action conveyed in concordance lines featuring the term ’app.’ This collaborative approach has shown its effectiveness in similar studies [152–154].

### Social actor representation

We underpin our use of Critical Discourse Analysis (CDA) with the Social Actor Representation (SAR) framework, which stems from Social Action Theory (SAT), to explore the interplay between grammatical and social agency. SAT, as proposed by [155], posits that human actions play a pivotal role in shaping society, institutions, and structures. This theoretical foundation provides valuable insights into human behaviour and societal dynamics, including the perceptions of ChatGPT users under our study. SAR delves into how grammatical structures, such as active or passive constructions and transitivity patterns, convey social agency by identifying the social actors within discourse [45]. An analysis of tweet syntax, sentence structures, and verb choices offers us a lens through which to gauge users’ perceptions regarding ChatGPT.

Key to the analysis of social actors are socio-semantic categories. These include ‘*excluding*’ agents, ‘*backgrounding*’ them, and the ‘*personalisation*’ or ‘*impersonalisation*’ of actors, often achieved through the use of agency metaphors, particularly relevant when considering non-human entities [57]. These linguistic features serve as markers of human-like perception and contribute to the attribution of responsibility [58]. Thus, using SAR is helpful when examining blame and responsibility in discourse.

In the realm of social actors, distinctions emerge between those representing a group (‘*genericised*’) or specific individuals (‘*specified*’). Actors can also fall into the categories of ‘*indetermined*’, as in cases like ‘someone,’ or ‘*determined*’, where their identity is explicitly known. These representation structures are illuminating in uncovering social and power dynamics within discourse, a concept demonstrated in other Twitter case studies applying CDA and SAR [156–158]. This study focuses solely on constructions where ChatGPT is the grammatical subject, rather than ones where it is the grammatical object, primarily due to the keyword in context within the concordance grids being ‘ChatGPT’.

Through our analysis of Twitter discourse, our primary objective is to uncover common themes in the presentation of the ChatGPT, thus shedding light on how power dynamics are conveyed within real-life data that pertains to algorithmic decisions, even when the operational mechanisms remain opaque. Additionally, we draw on prior research to identify semantically related thematic groups, contributing to a richer understanding of ChatGPT presentations and evolving perceptions over time [159, 160].

## Results

### Timeline overview of results

In our findings, we observed that the collocates ‘**be**’ and ‘**have**’ frequently co-occurred with ‘ChatGPT’ across all three time periods. Upon manual examination of tweets containing these combinations, the majority were identified as auxiliary verbs. In such instances, we treated them as multi-word expressions and analysed them based on their collective meaning, as they conveyed connections between agency and responsibility.

First, we looked at the frequency of active and passive verbal constructions including ‘ChatGPT’ to ascertain whether it was being presented as a social actor. This overview is shown in Table 2, where it features actively in 96% of the clauses. However, active and passive constructions alone do not necessarily provide a full account of how the app is presented in the discourse. For example, the app could be the subject of an active construction, yet could carry limited social agency. To avoid misinterpretations, we combined CL and CDA.

Each of these three time periods—launch, popularity, and developments—will be examined individually in this results section. A more comprehensive comparison between the periods occurs in the discussion section.

**Table 2:** Frequency of active and passive constructions of *ChatGPT*

Time period	Active	Passive	Total
Period 1: Launch (Nov and Dec 2022)	5160	212	5372
Period 2: Popularity (Jan 2023)	4346	135	4881
Period 3: Developments (Feb and Mar 2023)	5609	163	5772

### Time period 1: launch (November–December 2022)

#### Active constructions

**Table 3:** Top ten words ranked by collocational strength of ‘ChatGPT’+ R1 in November and December 2022

Rank	Collocate	Freq	Coll. freq.	logDice
1	be	3114	103,913	9.86593
2	have	408	24,494	8.80298
3	seem	76	1635	8.45962
4	do	203	15,667	8.29997
5	write	90	5978	8.01296
6	explain	48	1181	7.89235
7	give	51	3801	7.49875
8	make	54	6847	7.17016
9	know	42	4213	7.15571
10	generate	44	5279	7.07168

In November and December 2022, there were 5,160/5,372 instances of active constructions involving the app. The strongest 20 collocates are shown in Table 3.

‘**Have**’ (LogDice: 8.80298) was one of the strongest collocates. In tweets early in the discourse, ChatGPT is portrayed with a more active role, suggesting agency and engagement. For example, in one tweet, the author states that ‘ChatGPT has raised the alarm among educators’, indicating that it is actively involved in creating uncertainty and generating potential negative impact within the educational sphere. This agency metaphor suggests a more personalised and specific characterisation of ChatGPT, making it appear more like a social actor. However, in many instances, ChatGPT is presented as a tool, making use of verbal structures like ‘has been released’, ‘has been getting’, ‘has been fine-tuned’, and ‘has been trained’. These constructions position ChatGPT as the recipient of actions, rather than the one taking the actions. Therefore, these portrayals emphasise ChatGPT as an object or tool created and manipulated by external actors that are **excluded**. For instance, one user tweeted that ‘ChatGPT has been trained on a vast amount of text data’, which underscores ChatGPT’s passive role in training and highlights the human agency behind its development. Therefore, this use of the passive voice with ‘has been’ implies that ChatGPT is a tool or a product created and controlled by OpenAI, or perhaps other excluded entities, rather than an independent agent with decision-making capabilities.

The strong collocate ’**seem**’ (LogDice: 8.45962) may indicate uncertainty and speculation about ChatGPT’s capabilities, reflecting ongoing evaluation by users on Twitter. These tweets are subjective, based on individual experiences. It may also suggest that these are surface-level views, which, presently, do not have a strong evidence base to support them. The use of the verb ’**seem**’ in these tweets indicates that ChatGPT is perceived as more than just a neutral language model. For example, some tweets discuss ChatGPT’s capabilities, including its potential uses in various fields, such as education (‘ChatGPT seems like it can be a good education tool’), content generation (‘ChatGPT seems pretty good at completing short answer questions’), code writing (‘ChatGPT seems pretty good at writing, debugging, explaining, and translating code as well’), and its strengths and weaknesses compared to previous versions of GPT (‘ChatGPT seems to have a much smaller training set than GPT3’). The verb ’**seem**’ is usually used to express uncertainty or tentative observations about ChatGPT’s abilities. It implies that ChatGPT’s status as a capable social actor is not fully confirmed but is based on initial impressions.

Other tweets highlight ChatGPT’s responses to ethical, philosophical, or social questions (‘ChatGPT seems to pass a question on ethics, but fails when asked to apply it as a moral judgment’ and ‘ChatGPT seems to have the opinion that the laws of quantum mechanics are more fundamental than those of thermodynamics’), which suggests that users believe ChatGPT has a stance or opinion on these matters. This indicates that it is seen as more than just a neutral language model. Additionally, other tweets focus on users’ interactions with ChatGPT, including their experiences, questions, and opinions about using the model (‘ChatGPT seems to be able to generate.objs for simple stuff’ and ‘ChatGPT seems to be blowing my mind right now’). These tweets express users’ perceptions of ChatGPT’s behaviour and its responses to their queries, implying that its interactions with users are a key aspect of its social presence.

’**Do**’ (LogDice: 8.29997) is another strong collocate, often used to attribute actions or tasks to ChatGPT. This grammatical structure reinforces ChatGPT’s role as an active entity capable of carrying out tasks or functions. Many of these instances are examples of personalisation (‘ChatGPT did great, though’, ‘I’m really curious what ChatGPT does with the code you ask it to explain’). Equally, there are many negated constructions that state how much ChatGPT does not know (‘ChatGPT does not seem to have memory’, ‘ChatGPT does n ot know either what’s in the demo today’). In other instances, users express what ChatGPT is capable of (‘ChatGPT does surprisingly well on a Named Entity Recognition task’). Moreover, ’**do**’ is used to seek clarification or understanding about ChatGPT’s capabilities or limitations (‘What does ChatGPT do?’). This usage highlights users’ attempts to comprehend ChatGPT’s role. Occasionally, users compare ChatGPT to other entities or models, indicating differences in their abilities or functions (‘ChatGPT does not know about Dall’, ‘ChatGPT does not know anything about what happened after September 2021’), highlighting limitations in ChatGPT’s knowledge but still presenting it as a social actor capable of comprehension.

’**Write**’ (LogDice: 8.01296) is also strongly collocated with ’ChatGPT’. Tweets containing ’**do**’ mainly see ChatGPT depicted as actively writing content, algorithms, poems, and essays (‘I had @OpenAI’s ChatGPT write a backstory for my @moonbirds NFT’, ‘ChatGPT wrote monocular SLAM algorithm using GT-SAM’). It suggests an agency in producing textual or technical output, portraying ChatGPT as an active contributor to creative and informative endeavours. Moreover, on some occasions, ChatGPT is presented not just as a writer but as an influencer on creative content, highlighting its role in generating scripts, stories, and poems (‘ChatGPT [wrote] a script where Jesus is a C++ programmer from the 1990s’) and foregrounding its creative agency in shaping narratives (‘Not only did ChatGPT write us a script, but it also played the role of a DIRECTOR’). ChatGPT is also shown to play an active role in education by generating essays, research statements, and learning materials, all of which showcase it to be a social actor through *personalisation*.

‘**Explain**’ (LogDice: 7.89235) highlights ChatGPT’s role as an active participant in providing information and assistance. This choice of verb suggests that ChatGPT is not merely a passive tool but an entity that actively engages in discussions and offers explanations. For instance, one tweet states that ‘ChatGPT [explains]’ how its prompting system works. This tweet positions ChatGPT as a knowledgeable entity capable of explaining its own characteristics, demonstrating a degree of self-awareness and agency in the interaction through *personalisation*. Similarly, another tweet mentions that ChatGPT ‘explains the worst-case time complexity of the bubble sort algorithm’, highlighting its ability to provide detailed explanations on complex topics, further supporting the notion of it being a social actor. Overall, tweets with the collocate ‘**explain**’ depict ChatGPT as an active participant in conversations, engaging in explanations and demonstrating agency by offering information and insights on various subjects.

Similarly, tweets containing the collocate ‘**give**’ (LogDice: 7.49875) usually refer to ChatGPT actively providing information, advice, or responses to user queries, attributing agency. For instance, one tweet mentions that ‘ChatGPT [gives] purpose of life’, implying that ChatGPT has the capability to impart profound insights. Along with this, many other tweets underscore ChatGPT’s active role in supplying information, recommendations, or responses (‘ChatGPT gave me answers’, ‘ChatGPT gave me a list of advice’, and ‘ChatGPT gives me error messages’). As a result, these tweets highlight ChatGPT’s capacity to influence and engage with users through the act of providing, which is usually associated as a human attribute, thus *personalising* ChatGPT and portraying it as a social actor within the context of these conversations.

The collocate ‘**make**’ (LogDice: 7.17016) highlights ChatGPT’s role in creating content or influencing outcomes. For instance, one tweet author noted that ‘ChatGPT made a nice transcript’, emphasising its active content creation. Additionally, another tweet author questions if ‘ChatGPT made a mistake’ further highlighting ChatGPT’s capacity to not only produce results but also impact situations negatively, thus an example of *personalisation*. The use of ‘make’ portrays ChatGPT as a social actor involved in content generation and decision-making across various contexts (‘ChatGPT made me a content calendar’, ‘ChatGPT made the fry meme’), emphasising its role in cultural content creation. Thus, these presentations depict ChatGPT as a social actor capable of making, rather than ‘generating’, content.

The frequent occurrence of ‘**know**’ (LogDice: 7.15571) as a strong collocate of ChatGPT in these tweets highlights that it possesses knowledge, but it is more of an informational tool rather than an active social agent. For instance, one tweet author questions whether ‘ChatGPT knows C++‘ and, while this demonstrates ChatGPT’s capacity to provide information, this does not necessarily imply it as an independent actor in a social context. The use of ‘know‘ is consistently about the model’s ability to provide answers or information, rather than using or appropriating the knowledge. While these tweets suggest knowledge and capability, it does not inherently portray ChatGPT as a social actor with agency. Instead, it positions ChatGPT as a knowledge resource, a tool that can answer questions and provide information. In many instances, ChatGPT is presenting without overt agency, responding to queries and providing information based on pre-existing knowledge, as opposed to actively participating in social interactions or demonstrating agency. Therefore, these tweets do not strongly showcase ChatGPT as a social actor with significant agency; rather, they highlight its role as a tool for information retrieval.

’**Generate**’ (LogDice: 7.07168) is also a strong collocate. Despite being semantically similar to ’**write**’ and ‘**make**’, and still being used actively grammatically, the pragmatics of ’**generate**’ imply that ChatGPT’s function is to create content (‘ChatGPT generates code that uses the openAI go client’, ‘Don’t discount the @OpenAI Chatgpt generated content that results from better queries’), rather than it having any agential authority over the content it creates. Thus, its prowess as a social actor is limited in these tweets, as Twitter users are discussing ChatGPT as functional rather than *personalised*.

Through examining tweets containing the strongest collocate, ‘**be**’ (LogDice: 9.86593), there are many constructions that suggest a more passive and impersonalised characterisation of ChatGPT. In these tweets, ChatGPT is described as being ‘trained’ and ‘fine-tuned’. This grammatical structure often frames ChatGPT as a tool or product, rather than an active social actor with agency. For instance, the statement ‘ChatGPT was trained using Reinforcement Learning from Human Feedback’ positions ChatGPT as an outcome of a training process and lacks an active agency in the training, emphasising that ChatGPT is a result of a technical process rather than an independent social actor with responsibility.

#### Passive constructions

**Table 4:** Top ten words ranked by collocational strength of ‘by ChatGPT’ + L1 in November and December 2022

Rank	Collocate	Freq	Coll. freq.	logDice
1	Write	81	5978	8.73646
2	Inspire	5	191	8.55374
3	Hack	4	171	8.29956
4	Generate	49	5279	8.18321
5	Produce	9	820	8.11329
6	Amaze	3	196	7.80033
7	Edit	3	240	7.66312
8	Suggest	3	410	7.22961
9	Impress	3	463	7.11736
10	Create	16	4045	6.93324

The app is also the subject in passive constructions on 212/5372 occasions in the first time period, with collocates shown in Table 4. The strongest collocate was ‘**write**’ (LogDice: 8.73646). These passive constructions involving ‘**write**’ all attribute some degree of agency for the content creation to ChatGPT and portrays it as the author (‘Disclaimer: This tweet was written by ChatGPT’, ‘A thread about ChatGPT written by ChatGPT??’). This is very similar to ‘**generate**’ (LogDice: 8.18321), despite the slight semantic difference (‘The above response was generated by ChatGPT’, ‘The entire content of the book was generated by ChatGPT’). However, this attribution of authorship to ChatGPT raises questions about whether it is considered a social actor or a legitimate agent with autonomous decision-making capabilities (‘I have three job posts published, fully written by chatGPT’, ‘The canvas generated by ChatGPT’). While the tweets emphasise that the content is authored by ChatGPT, the passive voice implies that ChatGPT lacks personal initiative or intention in generating these tweets, *backgrounding* ChatGPT, in effect. This downplays the sense of agency and intent that is typically associated with human authors as it simultaneously elevates ChatGPT to the status of a content creator while undermining its role as a conscious social actor. Despite its passive presentation, ChatGPT is still discussed positively in these tweets.

In other tweets, verb choices like ‘**inspire**’ (LogDice: 8.55374), ‘**amaze**’ (LogDice: 7.80033) and ‘**impress**’ (LogDice: 7.11736) are often associated with humans or entities that possess intention and the capacity to influence or generate reactions. The grammatical structures featuring passive constructions tend to downplay ChatGPT’s agency in favor of emphasising the human response or intention, thus *backgrounding* ChatGPT. For example, one tweet author that states that they were ‘inspired by ChatGPT to create a language model chatbot’ places ChatGPT in a passive role, which is also enhanced by the fact that it is presented as serving as an inspiration, not as an active creator. Furthermore, the phrase ‘amazed by ChatGPT’ suggests that ChatGPT is the source of something astonishing, but it does not attribute this amazement directly to ChatGPT itself. This construction separates the source of amazement from the entity causing it.

### Time period 2: popularity (January 2023)

#### Active constructions

**Table 5:** Top ten words ranked by collocational strength of ‘ChatGPT’+ R1 in January 2023

Rank	Collocate	Freq	Coll. freq.	logDice
1	Be	2809	103,913	9.72852
2	Have	314	24,494	8.46688
3	Do	153	15,667	7.95154
4	Say	63	3771	7.94851
5	Pass	31	514	7.63791
6	Come	36	2522	7.3747
7	Write	51	5978	7.30556
8	Get	46	6290	7.1147
9	Make	44	6847	6.97852
10	Seem	22	1635	6.8565

In January 2023, there were 4,346 instances of active constructions involving the app. The strongest 20 collocates are shown in Table 5.

‘**Has**’ (LogDice: 8.46688) shows a slight decrease in collocational strength but remains one of the top collocates in January 2023. Once again, there are many occasions where ChatGPT is the object of constructions as they are presented in the passive voice (‘Chatgpt has been corrupted’, ‘ChatGPT has been trained on data till 2021’, ‘ChatGPT has been overhyped’). Within these examples, the subject of the construction is *excluded*, meaning that ChatGPT becomes the focus of the tweet despite having limited social agency. This demonstrates the further occurrence of ChatGPT disguising the human agents behind its creation and development, which may see it blamed or not trusted in the future.

‘**Do**’(LogDice: 7.95154) is, again, one of the strongest collocates, although slightly weaker in collocational strength in this time period comparatively. Similar to the first time period, negated constructions using ‘do’ to express ChatGPT’s limitations dominate the discourse (‘Even chat ChatGPT does not know why model Y is excluded from IRS’, ‘ChatGPT doesn’t reason, so it confidently makes self-contradictory assertions’), with users continuing to highlight what ChatGPT does not know or understand. Users still acknowledge ChatGPT as an active entity capable of carrying out, or not carrying out, tasks or functions, *personalising* it. However, in January 2023, tweets indicate a more nuanced understanding of ChatGPT’s capabilities in terms of performing specific actions or tasks, showing that users have perhaps become more specific in their queries or expectations. This may reflect an evolving understanding of what ChatGPT can do, which may impact how responsible or accountable it is.

Among the strong collocates is ‘**say**’ (LogDice: 7.94851), where tweets predominantly frame ChatGPT as a source of information and opinion. This is evident when users wrote ‘I use it sometimes to cross verify what ChatGPT says’ and ‘ChatGPT says No’. These expressions underscore its presence in facilitating conversations and potentially influencing individual decision-making Not only this, but tweets show ChatGPT’s potential to engage in dialogue as a mechanism for verifying or challenging information (‘ChatGPT says otherwise’ and ‘Here’s what ChatGPT said’). However, a recurring theme across these tweets is the notion of ChatGPT’s limited agency and capacity, as illuminated by statements like a recurrent acknowledgement of ChatGPT’s constrained autonomy (‘ChatGPT says it’s deleted—but with it in research mode’ and ‘ChatGPT says it is sorry and explains it is a language model’) underlines its primary function as a language model rather, which may limit its impact as a social actor.

Examining the collocate ‘**pass**’ (LogDice: 7.63791) reveals various dimensions of ChatGPT’s identity and functionality. Some tweets present ChatGPT as a wellspring of knowledge and expertise, attributing it the capability to pass exams and dispense specialised information (‘ChatGPT passed the US Medical Licensing Exam’, ‘ChatGPT passed its MBA final exam’, and ‘ChatGPT passed Bar Test’). These all indicate that ChatGPT possesses knowledge that is usually unique to humans only. However, there are instances where ChatGPT is depicted more as a tool or technology, lacking human-like agency. Some tweets humorously exaggerate its abilities, while others critically challenge its limitations. In this diverse discourse, the portrayal of ChatGPT ranges from an authoritative source of knowledge to a neutral instrument, reflecting multifaceted perceptions of its agency and social role.

‘**Make**’ (LogDice: 6.97852) signifies ChatGPT’s capacity to generate content. ChatGPT is portrayed as a creative force, with tweets acknowledging its capacity for content generation, facilitating easy solutions. Simultaneously, ’make’ is also used in the context of discussing ChatGPT’s power and influence, as it sparks both curiosity and engagement (‘ChatGPT makes students curious’). However, many of these tweets also focus on how ChatGPT generates false content (‘ChatGPT makes up fictitious titles of books’, ‘ChatGPT making up fake caselaw... yikes!’, and ‘ChatGPT making it too easy to generate some mock data’). While this may be undesirable to the tweet authors, this still portrays ChatGPT as a social actor with the capabilities to create something, whether that be true or false. Therefore, when comparing to the previous time period, a notable difference was the emphasis on ChatGPT generating false or fictitious content, depicting it as a social actor capable of producing both true and false information.

The collocate ‘**come**’ (LogDice: 7.3747) may imply an action associated with an entity that possesses the capacity to act and influence outcomes. However, the structure of the sentences frequently casts ChatGPT in a passive or reactive role rather than a proactive one. For instance, in one tweet, the phrase ‘CHATGPT came through for the rest’ suggests that ChatGPT acted responsively or supportively. This creates a sense of ChatGPT possessing some degree of control of its own emergence, hence *personalisation* with strong agency metaphor. While the use of ‘**come**’ attributes a degree of agency to ChatGPT as the entity performing the action, the overall structure of the sentences, and the specific contexts in which it is used tend to depict ChatGPT more as a responsive presence, rather than a proactive social actor. It portrays ChatGPT as a tool or resource that is called upon or utilised by humans, framing its agency within the boundaries set by the users.

Once again, ‘**write**’ (LogDice: 7.30556) is a strong collocate in January 2023. Grammatical structures indicate that ChatGPT is actively engaged in writing content and producing outputs. For instance, one tweet states, ‘@OpenAI’s ChatGPT wrote me a 2,000-word essay on global warming’, clearly attributing the act of writing to ChatGPT. Additionally, what users claim ChatGPT is writing appear to be more sophisticated than the tweets from November and December 2022, with recurrent patterns of attributing writing actions to ChatGPT in various contexts, including coding, content generation, poetry, and more. This includes instances where ChatGPT is held directly responsible for written work, exemplified by tweets such as ‘did ChatGPT write your abstract or not?’.

There are also many different contexts which the collocate ‘**get**’ (LogDice: 7.1147) is used. Tweets suggest that ChatGPT is capable of influencing or taking action. For instance, the tweet that discusses how ‘ChatGPT got me to try it’, this implies ChatGPT’s influence on the user, positioning it as a persuasive entity and, thus, portraying it as a social actor capable of prompting user actions. Similarly, ‘ChatGPT gets out of control’ in another tweet implies that ChatGPT can display certain behaviours or tendencies. It is also used as a synonym for understand (‘Even ChatGPT gets it’), alongside potentially negative impressions of understanding (‘ChatGPT got that wrong then’), further signifying its role as a social actor. This is similar to the varied usages of the collocate ‘**make**’ (LogDice: 6.97852), where, in some instances, ChatGPT is attributed with the ability to ‘make’ or influence decisions (‘ChatGPT makes not money’) and engage in humour (‘ChatGPT making fun of its own downtime is the new meta’). Tweets also discuss ChatGPT’s errors, saying that it ‘makes up references’ and ‘makes fictitious titles of books that don’t exist’.

Despite a lower collocational strength than the previous time period, ‘**seem**’ (LogDice: 6.8565) also appears as one of the top collocates for January 2023. Once again, tweets indicate that surface-level impressions of ChatGPT are being reported here. For example, it is stated in one tweet that ‘ChatGPT seems to be good at answering questions but not at asking them’, implying that these attributes are not definitively established. Similarly, ‘ChatGPT seems to fail in Comprehension test’ signifies that ChatGPT’s performance is subject to interpretation and not portrayed as a concrete action. The impression created by ‘**seem**’ is that users are reporting tentative impressions of ChatGPT, which limits its prowess as a social actor. However, this is not at the volume that it was in November and December 2022.

As in the previous discourse, many instances of ChatGPT collocating with ‘be’ (LogDice: 9.72852) are passive. Within this, tweet authors discuss how ChatGPT ‘was trained’ on various datasets’, ‘was fine-tuned on top of GPT$-$3.5’ and ‘is built on a Large Language Model’. One again, this foregrounds ChatGPT in a passive structure but *excludes* who did the training, fine-tuning or building, and shifting accountability.

#### Passive constructions

**Table 6:** Top ten words ranked by collocational strength of ‘by ChatGPT’ + L1 in January 2023

Rank	Collocate	Freq	Coll. freq.	logDice
1	Fascinate	3	64	8.7146
2	Replicate	3	88	8.57374
3	Generate	52	5279	8.28866
4	Disrupt	3	236	7.91963
5	Recommend	3	322	7.64245
6	Write	32	5978	7.4141
7	Impress	3	463	7.2789
8	Replace	5	965	7.17345
9	Produce	3	820	6.63368
10	Power	8	3186	6.28747

When examining passive constructions, shown in Table 6, similar passive presentations to the previous month can be seen. In many of these instances, ChatGPT was *backgrounded* in favor of a first-person account of an experience with ChatGPT. For example, tweets containing the semantically similar collocates ‘**fascinate**’ (LogDice: 8.7146) and ‘**impress**’ (LogDice: 7.2789) foreground the reaction from users to ChatGPT (‘The internet has become quickly fascinated by ChatGPT’, ‘am very impressed by ChatGPT’), rather than creating an active construction that places more agency with ChatGPT itself. Similarly for other collocates, such as ‘**replicate**’ (LogDice: 8.57374), ‘**disrupt**’ (LogDice: 7.91963) and ‘**recommend**’ (LogDice: 7.64245), tweet authors *backgrounded* the importance of the ChatGPT in the process, instead focusing on an evaluation of the content that the system can offer.

Alongside this, much like the previous time period, the verb ‘**generate**’ (LogDice: 8.28866) implies that ChatGPT may be actively creating content, but the passive construction places ChatGPT in the *background* (‘chatgpt is trained on the web circa 2021 I believe’), which may suggest that ChatGPT is merely a tool or mechanism that produces content without actively engaging in the creative process. ‘**Write**’ (LogDice: 7.4141) also implies the act of content creation but with a more deliberate and conscious effort, as per agency metaphor. However, in the passive construction, it still relegates ChatGPT to a *backgrounded* role (‘Check out this sample generated by ChatGPT‘), where the passive construction minimises ChatGPT’s agency in producing the sample and foregrounds the generated content. This is, therefore, seen to build on the previous month, where the ‘**backgrounding**’ of ChatGPT downplays its agency and intent, which are typically associated with human authors.

### Time period 3: developments (February–March 2023)

#### Active constructions

Between February and March 2023, 5,609 active presentations of ‘ChatGPT’ were found in the dataset collected, as shown in Table 7, numerous of which presented the app as a social actor.

**Table 7:** Top ten words ranked by collocational strength of ‘ChatGPT’ + R1 in February and March 2023

Rank	Collocate	Freq	Coll. freq.	logDice
1	Be	3234	103,913	9.91381
2	Have	468	24,494	8.97668
3	Do	252	15,667	8.57767
4	Announce	81	1103	8.55665
5	Say	77	3771	8.02049
6	Confirm	34	209	7.49992
7	Make	65	6847	7.3793
8	Give	48	3801	7.33422
9	Get	59	6290	7.3038
10	Write	56	5978	7.26577

‘**Has**’ (LogDice: 8.97668) is seen as the second strongest collocate again. Many presentations here show ChatGPT to be active, although there is a sharp increase in tweets with negated constructions. The tweets highlight that ChatGPT ‘has no profits’, ‘has no model of the world’, ‘has no humanity’, ‘has no moral thoughts’, ‘has no philosophical or moral reasoning’, ‘has no sentience’, and ‘has no long-term memory’. These phrasings collectively convey the idea that ChatGPT is devoid of qualities commonly ascribed to social actors, such as intention, consciousness, and ethical reasoning. Additionally, as seen previously, some active constructions still present ChatGPT passively (‘ChatGPT has been criticised by both the left and the right’, ‘ChatGPT has been banned by schools across the US’). ChatGPT here is seen as the recipient of criticism but does not imply that ChatGPT has any control or responsibility in this matter. These constructions imply that ChatGPT is a tool or resource subject to external decisions rather than a social actor with its own intentions, although it it still *foregrounded* in the tweet, while the subject of the constructions are either *backgrounded* or *excluded*.

As seen in the previous two time periods, many instances of the collocate ‘**do**’ (LogDice: 8.57767) are negated forms (‘ChatGPT does not have access to the whole internet’, ‘ChatGPT doesn’t model its own mental processes when it responds’, ‘ChatGPT does n’t have the actual knowledge or understanding’), highlighting ChatGPT’s limitations. There is still implied agency through *personalisation* in the majority of the cases (‘If ChatGPT does not find a way to share profits sooner than later’, ‘ChatGPT doesn’t let you make jokes about ants’), indicating that, despite its faults, ChatGPT remains a social actor due to its influence over a user. However, there appears to be more things that ChatGPT cannot do in these tweets compared to previous time periods. This may suggest that, despite the announcements from OpenAI about the advancements of ChatGPT, Twitter users are not seeing this reflected in their everyday use—either that, or their expectations for what ChatGPT can accomplish are becoming more realistic.

A verb previously unseen in the top ten strongest collocates for previous time periods is ‘**announce**’ (LogDice: 8.55665), where tweets portray ChatGPT to be an entity with agency, actively engaging in the act of conveying information to its audience. This portrayal perhaps aligns with the idea that ChatGPT has developed into a more proactive and socially engaged AI model. By choosing the word ‘announce,’ authors imply that ChatGPT is making intentional and purposeful actions, suggesting a level of agency (‘ChatGPT announces a paid model for $20/month’, ‘ChatGPT announced subscription plan’) The idea that ChatGPT itself has the capacity to reveal something, as opposed to OpenAI or determined humans involved, further portrays it as a social actor. This also marks a shift from prior time periods, where the choice of ’announce’ over other verbs like ‘share,’ ‘generate,’ or ‘produce’ positions ChatGPT as more than just a tool for answering questions; it suggests that it has the capacity to shape and communicate information in a way that aligns with the expectations of a social actor.

‘**Say**’ (LogDice: 8.02049) is seen as a strong collocate again, positioning ChatGPT as an entity capable of speech and portraying it as expressing opinions and providing information. The structure of these tweets reinforces ChatGPT as a social actor, allowing it to engage in conversations, make statements, and offer explanations. These tweets reflect ChatGPT as a conversational agent with the ability to convey information, even if this portrayal does not necessarily indicate human-like agency. Comparing this to the use of say’ in previous time periods, it becomes evident that the consistent use of ‘say’ with ChatGPT demonstrates an ongoing effort to present ChatGPT as an active and authoritative communicator. This could be a strategic choice by Twitter users to enhance its perceived reliability and credibility, as ’say’ implies certainty and a sense of authorship. In previous time periods, ‘**say**’ may have been used in a more general context, while the shift to in these tweets strengthens ChatGPT’s position as a conversational entity with agency.

However, there are also many instances of ‘say’ in this time period of users relaying a message from ChatGPT about its inability to access the most recent information (‘ChatGPT said: ’As an AI language model with a knowledge cut-off of 2021, I do not have access to real-time news updates”, ‘ChatGPT said ’As a language model AI, I do not have information about specific individuals unless it was mentioned and trained”). The increase in this type of reporting may be an expression of frustration by tweet authors, which could be seen as limiting the agency that ChatGPT has. Additionally, while ‘say’ is a clearly attributable to humans, the agency metaphor of the previously discussed ‘announce’ is higher.

The frequent use of ‘confirmed’ (LogDice: 7.49992) in tweets suggests a sense of verification or validation, making ChatGPT appear as an active participant in confirming information. While the term ‘confirm’ is typically associated with human actions, its use in relation to ChatGPT could imply a certain level of autonomy in assessing or affirming information, which might contribute to its portrayal as a social actor through *personalisation* (‘working with @OpenAI ChatGPT confirmed’, ‘ChatGPT confirmed to me that the social media algorithm isn’t rigged’). ‘Confirm’ does not feature in the top ten collocates for earlier time periods, which raises questions about why this particular structure is employed now and how it may impact the perception of ChatGPT as an active agent in the Twitter discourse. Tweets suggest that more users may be utilising ChatGPT to answer questions about itself, raising questions about the legitimacy of responses and the attribution of responsibility, accountability, and blame if something were to go wrong.

‘**Make**’ (LogDice: 7.3793) again appears as a strong collocate. In tweets, ‘**make**’ still portrays ChatGPT as a social actor with an agency, but there is a shift in the focus of its actions. The emphasis is on making technology and information accessible (‘ChatGPT made the tech accessible and approachable’) and facilitating specific actions (‘ChatGPT making strides all over the world’). This portrayal underscores ChatGPT’s role in improving access and convenience, signifying a shift to establishing it as a helpful entity or facilitator.

In some instances, the use of ‘**give**’ (LogDice: 7.33422) suggests that ChatGPT is a reliable source of information or answers, potentially emphasising a level of trustworthiness and certainly a degree of agency. For example, when one user wrote that ‘ChatGPT gave largely appropriate answers‘, it showcases ChatGPT to be in a position of providing valuable content, which might indicate a degree of accountability for the responses it generates. Conversely, the use of ’give’ also highlights situations where ChatGPT may generate incorrect or undesirable content (‘ChatGPT giving different calculation results’). This indicates that ChatGPT is not infallible, which could impact trust in its responses. Nevertheless, the agency is implied through *personalisation* again. This also sees a shift from earlier tweets that emphasised ChatGPT’s active role in providing information, attributing a significant level of agency to the model, toward a broader range of responses, including both positive and negative outcomes. While ChatGPT is still portrayed as an entity providing information or responses, there is an increased emphasis on the potential for ChatGPT to generate incorrect or undesirable content.

The examination of ‘**get**’ (LogDice: 7.3038) indicates mixed presentations of ChatGPT. Some tweets continue to attribute agency to ChatGPT, suggesting that ChatGPT can actively acquire or obtain things, like information, access, or data (‘ChatGPT gets subscription model with reliable access’). This portrays ChatGPT as an active and informed agent. In contrast, some tweets emphasise ChatGPT’s passive role in receiving information or data (‘ChatGPT got model training data set from Google and news’). This depicts ChatGPT as a recipient of data rather than an active agent obtaining it. In this context, ChatGPT is not seen as taking responsibility for obtaining data; instead, it seems to passively receive data from unspecified sources, *backgrounding* those who sourced the data. This variation in the use of ‘get’ might signify a shift in the portrayal of ChatGPT by Twitter users from an active and persuasive entity to a more passive recipient of information, with varying levels of agency and responsibility in different contexts.

‘**Write**’ (LogDice: 7.26577) still showcases ChatGPT as an active entity, and, to a certain degree, a social actor due to its ability to create content. However, the language used does not attribute intention, responsibility, or social agency to ChatGPT. For example, phrases like ‘ChatGPT writes code’ or ‘ChatGPT wrote this article for us’ focus on ChatGPT’s functionality as a text generation tool rather than its role as a conscious actor. The tweets provide instances of what ChatGPT is writing, and the content appears to be more sophisticated. Nevertheless, the degree of *personalisation* and the active influence on creative content and narratives are somewhat less prominent. Therefore, in these later tweets, the portrayal of ChatGPT’s agency is still evident, but it is not as strong as in the November and December 2022 tweets.

Along with the two previous periods, many of the constructions containing ‘**be**’ (LogDice: 9.91381) show ChatGPT to be the grammatical object. A focus of this appears to be how the LLM is trained to function (‘ChatGPT was trained with a reward model to be less toxic’, ‘ChatGPT was trained on a massive dataset of text from the internet’). This not only diminishes the sense of agency, portraying ChatGPT as more of a passive recipient of training, but it deliberately *excludes* who performed the training, making ChatGPT appear to be accountable and potentially blamed for incorrect information.

#### Passive constructions

**Table 8:** Top 11 words ranked by collocational strength of ‘by ChatGPT’ + L1 in February and March 2023

Rank	Collocate	Freq	Coll. freq.	logDice
1	Power	35	3186	8.39077
2	Pose	3	132	8.08114
3	Generate	45	5279	8.06402
4	Drive	3	265	7.63077
5	Write	33	5978	7.44425
6	Create	7	4045	5.74531
7	Tell	3	1608	5.74026
8	Provide	4	2302	5.69337
9	Use	19	15,715	5.28702
10	Give	4	3801	5.02272

Once again, as seen in Table 8, similar collocates to ‘by ChatGPT’ when compared to the previous time periods, can be seen. ‘**Power**’ (LogDice: 8.39077), which was featured as a strong collocate in both previous periods, becomes the strongest collocate. Here, representations underscore the passive construction of the sentences, which de-emphasises ChatGPT’s role and foregrounds other areas of interest. ChatGPT is deemed the source of power behind several applications (‘Microsoft Teams messaging is set to roll out a premium Team messaging powered by ChatGPT’, ‘Snapchat is releasing its own AI chatbot powered by ChatGPT’). The passive construction of these statements puts the focus on what is powered by ChatGPT, downplaying its agency.

Like previously, ‘**generate**’ (LogDice: 8.06402), ‘**write**’ (LogDice: 7.44425), and ‘**create**’ (LogDice: 5.74531) are strong collocates, positioning ChatGPT as the background tool or entity responsible for the content generation. In most of these instances, ChatGPT is depicted as a tool or mechanism for producing content, rather than as an active agent with agency (‘answers people’s questions with code generated by ChatGPT’, ‘Caption written by ChatGPT’, ‘Video title and description created by ChatGPT’). This framing of ChatGPT as a passive tool aligns with the idea of ChatGPT as a tool or instrument rather than a social actor.

‘**Pose**’ (LogDice: 8.08114) and ‘**drive**’ (LogDice: 7.63077 ) feature as collocates for the first time in this period. Here, tweets do not imply that ChatGPT actively poses a threat but rather that it is a passive entity with consequences (‘the threat posed by ChatGPT’), mitigating its impact as a social actor. Similarly, other tweets suggest that ChatGPT plays a role in driving the development of certain technologies, but it does not attribute agency or decision-making capabilities to ChatGPT itself due to the passive construction (‘R &D Boom Driven by ChatGPT’). Additionally, ‘tell’ (LogDice: 5.74026) and ‘provide’ (LogDice: 5.69337) are used to describe ChatGPT’s function of offering information and examples (‘I get to read stories told by ChatGPT’, ‘using the prompts provided by ChatGPT’). This underscores its role as a provider of information due to the passive presentations, foregrounding, once again, the human experience narrated in the tweets.

### Summary of results

These results point out that the app was primarily presented in an active manner, with 97% of occurrences (15,115 out of 15,625 constructions) falling into this category. However, it is important to note that some active presentations imbued the app with varying degrees of social agency. For example, approximately 1,514 presentations diminished this agency by mitigating activity, either through verb constructions or the inclusion of contextual information like ‘has been’ or ‘was developed’. As a result, ChatGPT was portrayed as a social actor in around 87% of the cases analyzed (13,601 out of 15,625).

Further examination reveals that, among the 13,601 active presentations where ChatGPT assumes the role of a social actor, three recurring themes emerge: ChatGPT creating, ChatGPT informing, and ChatGPT influencing. To address our research question, the ensuing discussion will delve into the connections between these themes and explore their relationship with the existing discourse and previous literature.

## Discussion

In this section, we will delve into our findings concerning the active and passive presentations. We will also address our research questions pertaining to Twitter users’ perceptions of ChatGPT’s agency, responsibility, accountability, trust, and blame. Our aim is to examine the evolution of these presentation trends within the discourse and establish their connections with prior research in the field. Additionally, we will explore the limitations of this study and provide recommendations for potential future research endeavours, taking these limitations into account.

### Trends of active agency

By conducting a comprehensive analysis of transitivity within the 15,625 concordance lines under consideration, and integrating the collocations of ‘ChatGPT’ and ‘by ChatGPT,’ along with a CDA-informed approach to agency and responsibility, supported by SAR, we have discerned three primary categories into which the active presentations of ChatGPT can be classified: creating, informing, and influencing. Therefore, the findings here reflect the social and political traits found by [32]. These categories collectively paint a picture of ChatGPT as both *personalised* [161] and capable of independent decision-making [44]. Through agency metaphor, ChatGPT is depicted as carrying out human-like actions [162]. Furthermore, the category of informing encompasses instances where ChatGPT acts autonomously as well as those where it simply operates as intended or designed. Notably, the first two categories encompass tweets where the app is either creating or informing effectively, but also encompass tweets where it is portrayed as not functioning as intended.

#### ChatGPT creating

The discourse around ChatGPT’s role as a social actor evolved somewhat in terms of ‘creating’ content. In the three time periods examined, ChatGPT’s role as a creator is presented as an active one, implying an agency similar to that of a human content creator. Although previous research indicates that humans may reject decision-making algorithms based if they are personified or anthropomorphised [82–85], this appears to not be the case here.

This portrayal developed over time, reflecting shifts in how ChatGPT is perceived in the online discourse. In the first time period (November to December 2022), Twitter users presented ChatGPT as a content creator with considerable agency. ChatGPT was depicted as actively ‘writing’ ‘generating’ and ‘making’ content, emphasising ChatGPT’s role as an independent creator, despite the strong collocate ‘seem’ indicating this was a surface-level impression only. Still, this positioned ChatGPT as an active agent, and its content was credited to ChatGPT itself. Despite the concerns that had been raised about ChatGPT’s agency [30, 110], this was not heavily present in the discourse. Here, the portrayal of ChatGPT as an active content creator might suggest an immediate trust in its ability to produce content effectively, despite occasional mentions of errors or undesired outputs.

The second time period considered (January–February 2023) witnessed a subtle evolution in the discourse regarding ChatGPT’s role as a content creator. While ChatGPT continued to be presented as an active entity, there was a shift in the focus of its creative agency. Content generation was still attributed to ChatGPT, but Twitter users placed an emphasis on ChatGPT pushing boundaries for more creative outputs, as well as making technology and information more accessible, in line with the previous findings [32–34]. In the third time period (February–March 2023), there was a slightly reduced emphasis on creative agency. While ChatGPT was still seen as ‘writing’ and ‘creating’ content, the language used often highlighted what ChatGPT was producing rather than its creative prowess. In these cases, ChatGPT’s role as an active content creator was evident, but the focus shifted toward the specific content produced rather than its creative capabilities. Perhaps, this highlights how, with increased transparency in the media regarding the limits of ChatGPT, fewer lines of agency are blurred [77, 79–81] Additionally, there is a renewed focus on the incorrect or undesirable content that ChatGPT is producing that is escalated from the previous time period, which links to prior research [112, 113]. This period still portrayed ChatGPT as a social actor, but with a more utilitarian and less creatively driven agency, perhaps implying that more human oversight to manage biases and errors is needed [117–119].

#### ChatGPT informing

The evolving theme of ChatGPT as an active communicator, marked by its role in ‘informing’, has implications for trust, accountability, and the perception of AI in information dissemination. ChatGPT’s role in informing is not novel within machine-generated systems, paralleling similar applications observed in automated news production within journalism studies [70–72]. In November and December 2022, ChatGPT is viewed as a resource that ‘provides’ or ‘gives’ information, with Twitter users acknowledging its capacity to answer questions and generate content, with only some recognising its limitations, such as the absence of access to real-time news updates. This rather nuanced understanding may reflect ChatGPT’s restricted knowledge base and capabilities. Despite this, many tweet authors still depict ChatGPT as a social actor, despite the origin of knowledge being withheld [20, 86].

As the discourse progressed into January 2023, there is a growing emphasis on ChatGPT ‘saying’ and ‘confirming’ information. It becomes increasingly portrayed as an active communicator that not only offers data but also validates it, with Twitter users positioning ChatGPT as a reliable source of information and relying on its outputs more blindly, despite advice against this by OpenAI. This is in line with the rationale for having a decision-making algorithm in the first place [1, 4–7]. The growing reliance on ChatGPT to confirm information may here signify an increasing trust in its veracity and accuracy, despite potential warnings against blind trust. As the discourse progressed further, ChatGPT is frequently depicted as ‘announcing’ information, reinforcing its role as a proactive communicator capable of making official statements and possessing human-like traits [32–34]. This progression reinforces the perception of ChatGPT as a social actor with the power to inform, yet also presents the dichotomy of ChatGPT also being depicted as an information source, despite the active grammatical presentation, due to agency metaphor [57, 58].

#### ChatGPT influencing

ChatGPT ‘influencing’ in the discourse underscores its profound impact on various facets of information sharing, decision-making and user engagement. The idea that ChatGPT can have any influence whatsoever could mean it possesses some agency regardless [50, 51]. Throughout the three time periods, Twitter users consistently depict ChatGPT as a social actor actively shaping and influencing the discourse and user experiences. For example, at the start of the discourse, ChatGPT is portrayed as an entity that ‘affects’ or ‘impacts’ user interactions and content. Twitter users describe how ChatGPT’s responses influence the discussions it engages in, often highlighting its potential to offer valuable information and influence user decisions, much like the findings from previous research [32–34]. However, unlike prior research, this influence is occasionally framed as a result of the tool’s functionality rather than its intentional actions.

As the discourse advances, ChatGPT is presented as an active entity that ‘helps’ and ‘shapes’ content, user opinions, and conversations. Users describe how ChatGPT plays a pivotal role in providing answers, clarifications, and solutions. Its influence is more apparent as it actively contributes to shaping the direction of conversations and offering valuable insights, indicating trust. In February and March 2023, the portrayal of ChatGPT’s influence becomes more nuanced as Twitter users emphasise its role in ‘guiding’ and ‘assisting’ users in various domains. This perhaps indicates a developing trust in being influenced by ChatGPT. This portrayal underscores ChatGPT’s active agency in shaping content and user experiences. The tweets do not, however, mention much about ChatGPT’s influence on job displacement [113].

#### Overarching depictions of active agency

The collection of these different representations demonstrates that Twitter users treated ChatGPT as a distinct social actor between November 2022 and March 2023—echoing the findings of [60, 68, 69] regarding the *personalisation* of AI more generally. This builds on existing research into the presentation of decision-making algorithms as social actors on social media sites [18]. Additionally, this gives further insight into how ChatGPT is presented as vacillating between creative actors and essentially unthinking tools, the findings associated with the research by [36]. This shift in how ChatGPT is presented on Twitter aligns with the ongoing debates about AI’s societal impact, encompassing benefits, challenges, biases, and ethical considerations [29, 114]. These results, however, do not necessarily support the findings from Dai et al., where ChatGPT was seen as an empowering tool for students’ epistemic agency [126]. Instead, throughout the discourse to varying degrees, it is presented as a content creation tool with potentially as much agency as a human, which may limit the agency of the human using it.

As mentioned previously, there were different variations in ChatGPT’s presentation—perhaps most notably, the tension between ChatGPT’s depiction as a creative social actor versus its presentation as an information source only. This was less consistent than perhaps anticipated, especially when examining collocates that may usually be associated with different degrees of agency metaphor. For example, the strong collocate ‘know’ in the first time period may, at first glance, indicate that ChatGPT is a social actor due to ‘know’ being indicative of a human attribute. However, after closer inspection of the tweets, it was being used in a manner that only presented ChatGPT as a knowledge resource, rather than possessing agency. In similar fashion, later in the discourse, the collocate ‘generate’, which may usually be associated with the semantic field of technology and imply less social agency, was used on occasions where ChatGPT was portrayed in a much more creative capacity. This inconsistent representation may mean that users were still uncertain of ChatGPT’s function and capabilities, and perhaps using language associated with ChatGPT and other generative AI technology when agency attribution was, in fact, much higher and replicated human interactions [94, 95].

The agency depicted in tweets may reflect a responsibility gap [15–17]. There are occasions in the discourse where the negative outcomes imply ChatGPT is being blamed [77, 96]. However, due to the dataset analysed only encompassing tweets published between November 2022 and March 2023, the discourse is perhaps not yet mature enough to see the long-term impact of some of these presentations.

### Passive presentations of ChatGPT

Despite the overwhelming number of active presentations in the discourse, the passive presentations of ChatGPT also contribute to a nuanced understanding of ChatGPT’s role as a social actor. These passive constructions tend to *background* ChatGPT [45] and *foreground* other aspects, largely the human experience, ultimately downplaying its agency [47]. This trend aligns with a perception of ChatGPT as more of a tool or mechanism than a social actor, which was also present in the active constructions.

For example, in November and December 2022, ChatGPT was presented passively as a tool used by Twitter users, with tweets indicating that ChatGPT was seen as a *backgrounded* tool that facilitates specific tasks. Here, the focus was on the output or outcome, downplaying the mechanism behind it and diminishing responsibility [56]. This presentation of ChatGPT continued into January 2023, with tweets highlighting ChatGPT’s role in powering or driving various technologies, positioning ChatGPT as somewhat of a driving force, yet in a passive and mechanistic way. These constructions suggest that ChatGPT’s role is more about providing the underlying technology than actively steering the direction of these developments. Finally, in February and March 2023, passive constructions underscored ChatGPT’s role in content creation but in a manner that foregrounded the content itself and not ChatGPT’s creative agency. This passive presentation reinforced the idea that ChatGPT serves as a tool for generating content, with less emphasis on its own creative intent. Occasionally, the passive constructions found that ChatGPT was being spoken about in a negative way, aligning with some of the observations made by Feier et al., who proposed that decision-making algorithms might deflect responsibility from more accountable individuals [20].

Overall, the passive presentations throughout the discourse demonstrate that ChatGPT is sometimes perceived by Twitter users as a behind-the-scenes facilitator rather than a proactive social actor. While ChatGPT’s agency is not entirely negated in these constructions, it is downplayed, and the focus is often on the human experience or the outcomes, technology, or content that ChatGPT contributes to, rather than ChatGPT’s own intentions or initiative. In this way, it could be seen that ChatGPT’s perhaps more as its passive role may imply less attribution of responsibility in certain contexts. The diminished agency in these passive depictions appeared to reduce its perceived impact [47, 56]. However, it is important to note that the passive presentations made up a relatively small proportion of the discourse.

### Ethical implications

The portrayal of ChatGPT holds ethical relevance in discussions about responsible AI deployment. Its dynamic representation, alternating between active agency and passive tool-like descriptions, mirrors the evolving societal understanding of AI’s role and the attributed responsibilities [163, 164]. This nuanced depiction aligns with ongoing debates on ethical considerations around AI’s agency, accountability, and its influence on user experiences [60, 165]. Moreover, contrasting perceptions of ChatGPT as both a creative force and a passive tool shed light on the complexities of AI’s agency and the potential ethical consequences involved [11, 63, 64]. The blurred lines between ChatGPT’s role as a decision-making entity and a facilitator of human tasks prompt inquiries into responsibility and accountability when AI contributes to both positive and negative outcomes, impacting trust and blame [15–17]..

This study’s focus on ChatGPT’s agency and its impact on user interactions and content creation provides insights into the ethical dimensions of AI’s societal integration [59]. Examining the degree of ChatGPT’s portrayal by Twitter users as a social actor reveals implicit links to broader ethical debates, encompassing AI’s involvement in decision-making, information dissemination, and its influence on society [18, 20].. In essence, while our study did not explicitly delve into ethical discourse per se, the findings highlight ethical considerations within the evolving perceptions of AI, exemplified by ChatGPT [42, 114, 120, 121].

### Limitations and future work

The methodological approach employed in this study addressed several challenges inherent in analysing a large dataset of 88,058 tweets, rendering manual examination unfeasible, as acknowledged in previous research [166]. To mitigate this challenge, CL techniques were utilised to filter the dataset and identify potential social actors. This approach involved analysing the keyword ‘ChatGPT’ and its collocates in the corpus, employing concordance grids and LogDice for examination. Despite not being infallible, this methodological choice aimed to overcome the impracticality posed by the sheer volume of tweets. Similarly, the potential subjective biases in interpreting instances of sarcasm and humour, particularly in tweets that were less explicit, was another limitation. This issue is not new [167, 168], but the combination of CDA with computationally aided techniques was employed to minimise the impact of this challenge and offers potential benefits for future research as well.

In this study, the focus on the keyword ‘ChatGPT’ led to the exclusion of cases where the app was described without the explicit use of the term itself. Future research could explore these explicitly excluded constructions to gain a more comprehensive understanding of the discourse surrounding ChatGPT, especially exploring other potential social actors, such as OpenAI. Additionally, it is worth considering that ChatGPT may have been discussed in tweets without specific reference to its name. Exploring synonyms for ‘ChatGPT’ in this specific context, such as ‘LLM’, ‘application’ or ‘tool’, might offer insights into other synonyms for social actors replacing the app.

The brevity of Twitter discourse, primarily limited to a 280-character count at the time of analysis, may have also influenced the predominantly active presentation of ChatGPT to facilitate conciseness. For instance, ‘ChatGPT wrote my essay’ (19 characters) was more concise than ‘my essay was written by ChatGPT’ (26 characters) while conveying the same semantic meaning. This increased prevalence of active presentations likely impacted the instances where ChatGPT was clearly presented as a social actor. As a result, future research might involve examining social media or text-sharing platforms with different character limits to assess the prevalence of active presentations implying social actor status.

Finally, one of the main limitations of the study is that the dataset only encompasses tweets published in the ‘hype period’ between November 2022 and March 2023, meaning that the discourse surrounding ChatGPT online will have likely evolved in different ways since this time period. This provides motivation to explore whether there have been alterations or developments in the depiction of ChatGPT as a social actor on Twitter by examining more contemporary tweets.

## Conclusion

Based on our comprehensive linguistic analysis, combining CL, CDA and SAR, within the dataset of tweets containing the term ‘ChatGPT’ from November 2022 to March 2023, it is evident that Twitter users overwhelmingly depicted ChatGPT as a social actor, albeit with varying degrees of social agency. The active presentation of ChatGPT was prevalent in approximately 86% of cases, often achieved through linguistic techniques like *personalisation* and *agency metaphor*. These active portrayals predominantly highlighted ChatGPT’s roles in content creation, information dissemination, and influence, signaling a growing reliance on and trust in its outputs. Furthermore, our analysis unveiled a dynamic portrayal of ChatGPT, vacillating between a creative social actor and an information source, potentially mirroring user uncertainty regarding its capabilities.

Despite a majority of cases portraying ChatGPT as a social actor, it was also presented passively. In such instances, ChatGPT was often *backgrounded* in order to *foreground* the human experience, the developers or other issues, downplaying its agency and portraying it more as a tool or mechanism than a proactive actor. This reduced agency in passive depictions seems to diminish ChatGPT’s perceived impact in certain contexts, although this was seen in only 14% of occasions and fell proportionally as the discourse continued.

Several implications stem from our findings. First, the nuanced understanding of how the social agency of decision-making algorithms is constructed in online discourses can be valuable for both AI developers and policymakers in gauging public perceptions and expectations, especially when it comes to trust and blame. Additionally, while ChatGPT is often portrayed as a social actor, there is still variation in how it is presented. Some users saw it as a tool or mechanism, which may have implications for responsibility and accountability. Understanding how different presentations influence perceptions of responsibility is essential, particularly in cases where AI systems have real-world impacts. Finally, as our findings show that ChatGPT’s role in ‘informing’ and ‘influencing’ is emphasised on Twitter, this implies that users may rely on ChatGPT for information and trust its outputs. This has implications for information dissemination and trust in AI-generated content, where developers and promoters of these systems may consider these findings to improve user experiences and maintain trust.

In summary, this study sheds light on the dynamics of social agency as conveyed through public discourse on social media concerning algorithmic-operated decisions, especially when the AI’s agency remains hidden. This relationship, exemplified in the context of ChatGPT, grammatical agency, and social agency, builds on prior research on the social agency of decision-making algorithms [18, 60, 61, 65, 82]. Hence, our investigation significantly adds to the understanding of the social impact of ChatGPT, employing a combination of CL and CDA, underpinned by SAR. In essence, our findings suggest that social media discourse often portrays ChatGPT as possessing human-like agency, influencing aspects like responsibility, trust, and attributions of blame.

## Data Availability

All data generated or analysed during this study are included in this published article and its supplementary information files.

## References

[1] Wagner, B.: Liable, but not in control? Ensuring meaningful human agency in automated decision-making systems. Policy Internet **11**(1), 104–122 (2019)

[2] Pepper, C., Reyes-Cruz, G., Pena, A.R., Dowthwaite, L., Babbage, C.M., Wagner, H., Nichele, E., Fischer, J.E., et al.: Understanding trust and changes in use after a year with the NHS COVID-19 contact tracing app in the United Kingdom: longitudinal mixed methods study. J. Med. Internet Res. **24**(10), 40558 (2022) 10.2196/40558 PMC9578414 36112732

[3] Araujo, T., Helberger, N., Kruikemeier, S., De Vreese, C.H.: In AI we trust? Perceptions about automated decision-making by artificial intelligence. AI Soc. **35**(3), 611–623 (2020)

[4] Busch, P.A., Henriksen, H.Z.: Digital discretion: a systematic literature review of ict and street-level discretion. Inf. Polity **23**(1), 3–28 (2018)

[5] Panagiotopoulos, P., Klievink, B., Cordella, A.: Public Value Creation in Digital Government. Elsevier (2019)

[6] Bullock, J.B.: Artificial intelligence, discretion, and bureaucracy. Am. Rev. Public Admin. **49**(7), 751–761 (2019)

[7] Young, M.M., Bullock, J.B., Lecy, J.D.: Artificial discretion as a tool of governance: a framework for understanding the impact of artificial intelligence on public administration. Perspect. Public Manag. Gov. **2**(4), 301–313 (2019)

[8] Crang, M., Graham, S.: Sentient cities ambient intelligence and the politics of urban space. Inf. Commun. Soc. **10**(6), 789–817 (2007)

[9] Ziewitz, M.: Governing algorithms: myth, mess, and methods. Sci. Technol. Hum. Values **41**(1), 3–16 (2016)

[10] Bonnefon, J.-F., Shariff, A., Rahwan, I.: The social dilemma of autonomous vehicles. Science **352**(6293), 1573–1576 (2016) 27339987 10.1126/science.aaf2654

[11] Floridi, L., Cowls, J., Beltrametti, M., Chatila, R., Chazerand, P., Dignum, V., Luetge, C., Madelin, R., Pagallo, U., Rossi, F., et al.: AI4people-an ethical framework for a good ai society: opportunities, risks, principles, and recommendations. Mind. Mach. **28**, 689–707 (2018) 10.1007/s11023-018-9482-5 PMC6404626 30930541

[12] Coates, D.J., Tognazzini, N.A.: Blame: Its Nature and Norms. Oxford University Press (2013)

[13] Baumeister, R.F.: Evil: Inside Human Cruelty and Violence. WH Freeman/Times Books/Henry Holt & Co (1996)

[14] Ross, L.: The intuitive psychologist and his shortcomings: distortions in the attribution process. In: Advances in Experimental Social Psychology, vol. 10, pp. 173–220. Elsevier (1977)

[15] Mittelstadt, B.D., Allo, P., Taddeo, M., Wachter, S., Floridi, L.: The ethics of algorithms: mapping the debate. Big Data Soc. **3**(2), 2053951716679679 (2016)

[16] Tollon, F.: Responsibility gaps and the reactive attitudes. AI Ethics **3**(1), 295–302 (2023)

[17] Munch, L., Mainz, J., Bjerring, J.C.: The value of responsibility gaps in algorithmic decision-making. Ethics Inf. Technol. **25**(1), 21 (2023)

[18] Heaton, D., Nichele, E., Clos, J., Fischer, J.E.: “The algorithm will screw you’’: blame, social actors and the 2020 A Level results algorithm on Twitter. PLoS ONE **18**(7), 0288662 (2023) 10.1371/journal.pone.0288662 PMC10370707 37494323

[19] Olhede, S., Wolfe, P.J.: Blame the algorithm? Significance **17**(5), 12–12 (2020)

[20] Feier, T., Gogoll, J., Uhl, M.: Hiding behind machines: when blame is shifted to artificial agents. CoRR **abs/2101.11465** (2021). arXiv:2101.11465

[21] Peeters, R.: The agency of algorithms: understanding human-algorithm interaction in administrative decision-making. Inf. Polity **25**(4), 507–522 (2020)

[22] Velkova, J., Kaun, A.: Algorithmic resistance: media practices and the politics of repair. Inf. Commun. Soc. **24**(4), 523–540 (2021)

[23] Hariri, W.: Unlocking the potential of ChatGPT: a comprehensive exploration of its applications. Technology **15**(2), 16 (2023)

[24] Rathore, B.: Future of AI and generation alpha: ChatGPT beyond boundaries. Eduzone **12**(1), 63–68 (2023)

[25] Firat, M.: How ChatGPT can transform autodidactic experiences and open education. Open Education Faculty, Anadolu Unive, Department of Distance Education (2023)

[26] Zhuo, T.Y., Huang, Y., Chen, C., Xing, Z.: Exploring AI ethics of ChatGPT: a diagnostic analysis. arXiv preprint arXiv:2301.12867 (2023)

[27] Kirmani, A.R.: Artificial intelligence-enabled science poetry. ACS Energy Lett. **8**, 574–576 (2022)

[28] Ye, R.: The power of prompting: navigating the future of AI and machine learning. Rizwan Ye (2023)

[29] Abdullah, M., Madain, A., Jararweh, Y.: ChatGPT: fundamentals, applications and social impacts. In: 2022 Ninth International Conference on Social Networks Analysis, Management and Security (SNAMS), IEEE, pp. 1–8 (2022)

[30] Verma, P., Lerman, R.: What is ChatGPT? Everything you need to know about chatbot from OpenAI. WP Company (2023)

[31] Rogers, E.M.: Diffusion of innovations: modifications of a model for telecommunications. Die diffusion von innovationen in der telekommunikation, pp. 25–38 (1995)

[32] Al Lily, A.E., Ismail, A.F., Abunaser, F.M., Al-Lami, F., Abdullatif, A.K.A.: ChatGPT and the rise of semi-humans. Humanit Soc. Sci. Commun. **10**(1), 1–12 (2023)

[33] Choudhury, A., Shamszare, H.: Investigating the impact of user trust on the adoption and use of ChatGPT: survey analysis. J. Med. Internet Res. **25**, 47184 (2023) 10.2196/47184 PMC10337387 37314848

[34] Sundar, S.S., Liao, M.: Calling BS on ChatGPT: reflections on AI as a communication source. J. Commun. Monogr. **25**(2), 165–180 (2023)

[35] Gutiérrez, J.L.M.: On actor-network theory and algorithms: ChatGPT and the new power relationships in the age of AI. AI Ethics, 1–14 (2023)

[36] Bran, E., Rughiniş, C., Nadoleanu, G., Flaherty, M.G.: The emerging social status of generative AI: vocabularies of ai competence in public discourse. In: 2023 24th International Conference on Control Systems and Computer Science (CSCS), IEEE, pp. 391–398 (2023)

[37] Shijie, S., Yuxiang, Z., Qinghua, Z.: From eliza to ChatGPT: AI-generated content (AIGC) credibility evaluation in human-intelligent interactive experience. Inf. Doc. Serv. **44**(4), 35–42 (2023)

[38] Taecharungroj, V.: “What can ChatGPT do?’’ Analyzing early reactions to the innovative AI chatbot on Twitter. Big Data Cognit. Comput. **7**(1), 35 (2023)

[39] Korkmaz, A., Aktürk, C., Talan, T.: Analyzing the user’s sentiments of ChatGPT using twitter data. Iraqi J. Comput. Sci. Math. **4**(2), 202–214 (2023)

[40] Weller, K., Bruns, A., Burgess, J., Mahrt, M., Puschmann, C.: Twitter and Society. Peter Lang, New York (2013)

[41] McCormick, T.H., Lee, H., Cesare, N., Shojaie, A., Spiro, E.S.: Using Twitter for demographic and social science research: tools for data collection and processing. Soc. Methods Res. **46**(3), 390–421 (2017) 10.1177/0049124115605339 PMC5639727 29033471

[42] Haque, M.U., Dharmadasa, I., Sworna, Z.T., Rajapakse, R.N., Ahmad, H.: “I think this is the most disruptive technology”: exploring sentiments of ChatGPT early adopters using Twitter data. arXiv preprint arXiv:2212.05856 (2022)

[43] Leiter, C., Zhang, R., Chen, Y., Belouadi, J., Larionov, D., Fresen, V., Eger, S.: ChatGPT: a meta-analysis after 2.5 months. arXiv preprint arXiv:2302.13795 (2023)

[44] Richardson, P., Mueller, C.M., Pihlaja, S.: Cognitive Linguistics and Religious Language: An Introduction. Routledge (2021)

[45] Van Leeuwen, T.: Discourse and Practice: New Tools for Critical Discourse Analysis. Oxford University Press (2008)

[46] Leslie, A.M.: A Theory of Agency. Citeseer (1993)

[47] Clark, W.R.: Agents and structures: two views of preferences, two views of institutions. Int. Stud. Quart. **42**(2), 245–270 (1998)

[48] Gallagher, S.: Philosophical conceptions of the self: implications for cognitive science. Trends Cogn. Sci. **4**(1), 14–21 (2000) 10637618 10.1016/s1364-6613(99)01417-5

[49] Silver, C.A., Tatler, B.W., Chakravarthi, R., Timmermans, B.: Social agency as a continuum. Psychon. Bull. Rev. **28**(2), 434–453 (2021) 33289061 10.3758/s13423-020-01845-1 PMC8062427

[50] Bandura, A.: Social cognitive theory: an agentic perspective. Annu. Rev. Psychol. **52**(1), 1–26 (2001) 11148297 10.1146/annurev.psych.52.1.1

[51] Giddens, A.: The Constitution of Society: Outline of the Theory of Structuration, vol. 349. University of California Press (1986)

[52] Arnett, J.J.: The neglected 95%: why American psychology needs to become less American. Am. Psychol. **63**(7), 602–614 (2016) 10.1037/0003-066X.63.7.602 18855491

[53] Marková, I.: Dialogicality and Social Representations: The Dynamics of Mind. Cambridge University Press (2003)

[54] Zimmerman, B.J.: Self-efficacy: an essential motive to learn. Contemp. Educ. Psychol. **25**(1), 82–91 (2000) 10620383 10.1006/ceps.1999.1016

[55] Oktar, L.: The ideological organization of representational processes in the presentation of us and them. Discourse Soc. **12**(3), 313–346 (2001)

[56] Comrie, B.: In defense of spontaneous demotion: the impersonal passive. In: Grammatical Relations, pp. 47–58. Brill (1977)

[57] Morris, M.W., Sheldon, O.J., Ames, D.R., Young, M.J.: Metaphors and the market: consequences and preconditions of agent and object metaphors in stock market commentary. Organ. Behav. Hum. Decis. Process. **102**(2), 174–192 (2007)

[58] Tourish, D., Hargie, O.: Metaphors of failure and the failures of metaphor: a critical study of root metaphors used by bankers in explaining the banking crisis. Organ. Stud. **33**(8), 1045–1069 (2012)

[59] Heaton, D., Clos, J., Nichele, E., Fischer, J.E.: The social impact of decision-making algorithms: reviewing the influence of agency, responsibility and accountability on trust and blame. In: Proceedings of the First International Symposium on Trustworthy Autonomous Systems, pp. 1–11 (2023)

[60] Rubel, A., Castro, C., Pham, A.: Algorithms, agency, and respect for persons. Soc. Theory Pract. **46**(3), 547–572 (2020)

[61] Lamanna, C., Byrne, L.: Should artificial intelligence augment medical decision making? The case for an autonomy algorithm. AMA J. Ethics **20**(9), 902–910 (2018) 10.1001/amajethics.2018.902 30242824

[62] Bryson, J.J.: The Artificial Intelligence of the Ethics of Artificial Intelligence, vol. 1. The Oxford Handbook of Ethics of AI (2020)

[63] Holford, W.D.: ‘Design-for-responsible’algorithmic decision-making systems: a question of ethical judgement and human meaningful control. AI Ethics **2**(4), 827–836 (2022)

[64] Turton, W.: The algorithm is innocent. Google and Facebook deflect responsibility onto algorithms, as if they don’t control their own code. The Outline (2017)

[65] Zarsky, T.: The trouble with algorithmic decisions: an analytic road map to examine efficiency and fairness in automated and opaque decision making. Sci. Technol. Hum. Values **41**(1), 118–132 (2016)

[66] Barocas, S., Selbst, A.D.: Big data’s disparate impact. Calif. Law Rev. **104**, 671 (2016)

[67] Bodo, B., Helberger, N., Irion, K., Zuiderveen Borgesius, F., Moller, J., Velde, B., Bol, N., Es, B., Vreese, C.: Tackling the algorithmic control crisis-the technical, legal, and ethical challenges of research into algorithmic agents. Yale JL Technol. **19**, 133 (2017)

[68] Reeves, B., Nass, C.: The media equation: how people treat computers, television, and new media like real people. Cambridge (UK) **10**, 10 (1996)

[69] Sundar, S.S.: Rise of machine agency: a framework for studying the psychology of human-AI interaction (HAII). J. Comput. Mediat. Commun. **25**(1), 74–88 (2020)

[70] Graefe, A., Bohlken, N.: Automated journalism: a meta-analysis of readers’ perceptions of human-written in comparison to automated news. Media Commun. **8**(3), 50–59 (2020)

[71] Dörr, K.N.: Mapping the field of algorithmic journalism. Digit. J. **4**(6), 700–722 (2016)

[72] Clerwall, C.: Enter the robot journalist: users’ perceptions of automated content. In: The Future of Journalism: in an Age of Digital Media and Economic Uncertainty, pp. 165–177. Routledge (2017)

[73] Riegler, C.: The moral decision-making capacity of self-driving cars: socially responsible technological development, algorithm-driven sensing devices, and autonomous vehicle ethics. Contemp. Read. Law Soc. Just. **11**, 15 (2019)

[74] Clark, J.R., Large, D.R., Shaw, E., Nichele, E., Trigo, M.J.G., Fischer, J.E., Burnett, G., Stanton, N.A.: Identifying interaction types and functionality for automated vehicle virtual assistants: an exploratory study using speech acts cluster analysis. Appl. Ergon. **114**, 104152 (2024) 37856899 10.1016/j.apergo.2023.104152

[75] Beer, D.: The Social Power of Algorithms. Taylor and Francis (2017)

[76] Meisner, C., Duffy, B.E., Ziewitz, M.: The Labor of Search Engine Evaluation: Making Algorithms More Human or Humans More Algorithmic? New Media and Society (2022)

[77] Burrell, J.: How the machine ‘thinks’: understanding opacity in machine learning algorithms. Big Data Soc. **3**(1), 2053951715622512 (2016)

[78] Felzmann, H., Villaronga, E.F., Lutz, C., Tamò-Larrieux, A.: Transparency you can trust: transparency requirements for artificial intelligence between legal norms and contextual concerns. Big Data Soc. **6**(1), 2053951719860542 (2019)

[79] Pasquale, F.: The Black Box Society: The Secret Algorithms that Control Money and Information. Harvard University Press (2015)

[80] Diakopoulos, N.: Accountability in algorithmic decision making. Commun. ACM **59**(2), 56–62 (2016)

[81] Selbst, A.D., Boyd, D., Friedler, S.A., Venkatasubramanian, S., Vertesi, J.: Fairness and abstraction in sociotechnical systems. In: Proceedings of the Conference on Fairness, Accountability, and Transparency, pp. 59–68 (2019)

[82] Mahmud, H., Islam, A.N., Ahmed, S.I., Smolander, K.: What influences algorithmic decision-making? A systematic literature review on algorithm aversion. Technol. Forecast. Soc. Chang. **175**, 121390 (2022)

[83] Yu, H.: A cogitation on the ChatGPT craze from the perspective of psychological algorithm aversion and appreciation. Psychology Research and Behavior Management, pp. 3837–3844 (2023) 10.2147/PRBM.S430936 PMC10505389 37724135

[84] Schoenherr, J.R., Abbas, R., Michael, K., Rivas, P., Anderson, T.D.: Designing AI using a human-centered approach: explainability and accuracy toward trustworthiness. IEEE Trans. Technol. Soc. **4**(1), 9–23 (2023)

[85] Waddell, T.F.: Can an algorithm reduce the perceived bias of news? Testing the effect of machine attribution on news readers’ evaluations of bias, anthropomorphism, and credibility. J. Mass Commun. Q. **96**(1), 82–100 (2019)

[86] Grange, C.: Algorithmically controlled automated decision-making and societal acceptability: Does algorithm type matter? In: HICSS, pp. 1–10 (2022)

[87] Asatiani, A., Malo, P., Nagbøl, P.R., Penttinen, E., Rinta-Kahila, T., Salovaara, A.: Sociotechnical envelopment of artificial intelligence: an approach to organizational deployment of inscrutable artificial intelligence systems. J. Assoc. Inf. Syst. (JAIS) **22**(2), 252–352 (2021)

[88] Ford, M.: Architects of Intelligence: The Truth About AI from the People Building it. Packt Publishing Ltd (2018)

[89] Murad, M.: Beyond the“ black box”: Enabling meaningful transparency of algorithmic decision-making systems through public registers. PhD thesis, Massachusetts Institute of Technology (2021)

[90] Nishant, R., Schneckenberg, D., Ravishankar, M.: The formal rationality of artificial intelligence-based algorithms and the problem of bias. J. Inf. Technol. (2023). 10.1177/02683962231176842

[91] Prinz, W.: Open Minds: The Social Making of Agency and Intentionality. MIT Press (2012)

[92] Xi, Z., Chen, W., Guo, X., He, W., Ding, Y., Hong, B., Zhang, M., Wang, J., Jin, S., Zhou, E., et al.: The rise and potential of large language model based agents: a survey. arXiv preprint arXiv:2309.07864 (2023)

[93] Carlson, M.: Automating judgment? Algorithmic judgment, news knowledge, and journalistic professionalism. New Media Soc. **20**(5), 1755–1772 (2018)

[94] Petrović, V.M.: Artificial intelligence and virtual worlds-toward human-level AI agents. IEEE Access **6**, 39976–39988 (2018)

[95] Lepri, B., Oliver, N., Letouzé, E., Pentland, A., Vinck, P.: Fair, transparent, and accountable algorithmic decision-making processes: the premise, the proposed solutions, and the open challenges. Philos. Technol. **31**, 611–627 (2018)

[96] Jobin, A., Ienca, M., Vayena, E.: The global landscape of ai ethics guidelines. Nat. Mach. Intell. **1**(9), 389–399 (2019)

[97] Ray, P.P.: ChatGPT: a comprehensive review on background, applications, key challenges, bias, ethics, limitations and future scope. Internet Things Cyber-Phys. Syst. **3**, 121–154 (2023)

[98] Hassani, H., Silva, E.S.: The role of ChatGPT in data science: how AI-assisted conversational interfaces are revolutionizing the field. Big Data Cogn. Comput. **7**(2), 62 (2023)

[99] Duarte, F.: Number of CHATGPT users (Dec 2023). Exploding topics (2023). https://explodingtopics.com/blog/chatgpt-users

[100] Haleem, A., Javaid, M., Singh, R.P.: An era of ChatGPT as a significant futuristic support tool: a study on features, abilities, and challenges. Bench Council Trans. Benchmarks Stand. Eval. **2**(4), 100089 (2022)

[101] Whalen, J., Mouza, C., et al.: ChatGPT: challenges, opportunities, and implications for teacher education. Contemp. Issues Technol. Teach. Educ. **23**(1), 1–23 (2023)

[102] Ali, R., Tang, O.Y., Connolly, I.D., Fridley, J.S., Shin, J.H., Zadnik Sullivan, P.L., Cielo, D., Oyelese, A.A., Doberstein, C.E., Telfeian, A.E., et al.: Performance of ChatGPT, GPT-4, and Google Bard on a neurosurgery oral boards preparation question bank. medRxiv, 2023–04 (2023) 10.1227/neu.0000000000002551 37306460

[103] Zhang, B.: Chatgpt, an opportunity to understand more about language models. Med. Ref. Serv. Q. **42**(2), 194–201 (2023) 37104260 10.1080/02763869.2023.2194149

[104] Dönmez, İ, Sahin, I., Gülen, S.: Conducting academic research with the AI interface ChatGPT: challenges and opportunities. J. STEAM Educ. **6**(2), 101–118 (2023)

[105] Antaki, F., Touma, S., Milad, D., El-Khoury, J., Duval, R.: Evaluating the performance of chatgpt in ophthalmology: an analysis of its successes and shortcomings. Ophthalmol. Sci. **10**, 100324 (2023) 10.1016/j.xops.2023.100324 PMC10272508 37334036

[106] Aiyappa, R., An, J., Kwak, H., Ahn, Y.-Y.: Can we trust the evaluation on ChatGPT? arXiv preprint arXiv:2303.12767 (2023)

[107] Cao, Y., Zhai, J.: Bridging the gap-the impact of chatgpt on financial research. J. Chin. Econ. Bus. Stud. **5**, 1–15 (2023)

[108] Sanderson, K.: Gpt-4 is here: what scientists think. Nature **615**(7954), 773 (2023) 36928404 10.1038/d41586-023-00816-5

[109] Roose, K.: GPT-4 is exciting and scary. The New York Times (2023). https://www.nytimes.com/2023/03/15/technology/gpt-4-artificial-intelligence-openai.html

[110] Kelly, S.M.: This AI chatbot is dominating social media with its frighteningly good essays | CNN business. Cable News Network (2023). https://edition.cnn.com/2022/12/05/tech/chatgpt-trnd/index.html

[111] Kocaballi, A.B.: Conversational ai-powered design: Chatgpt as designer, user, and product. arXiv preprint arXiv:2302.07406 (2023)

[112] Aljanabi, M.: ChatGPT: future directions and open possibilities. Mesop. J. Cybersecur. **2023**, 16–17 (2023)

[113] Najafali, D., Camacho, J.M., Reiche, E., Galbraith, L., Morrison, S.D., Dorafshar, A.H.: Truth or lies? The pitfalls and limitations of chatgpt in systematic review creation. Aesthet. Surg. J. **093**, 5 (2023) 10.1093/asj/sjad093 PMC10349645 37018119

[114] Zhou, J., Müller, H., Holzinger, A., Chen, F.: Ethical chatgpt: Concerns, challenges, and commandments. arXiv preprint arXiv:2305.10646 (2023)

[115] Dowthwaite, L., Fischer, J., Perez, E., Portillo, V., Nichele, E., Goulden, M., McAuley, D.: Public adoption and trust in the Covid-19 contact tracing app in the UK: A survey study (preprint). J. Med. Internet Res. (2021). 10.2196/29085 10.2196/29085 PMC8451731 34406960

[116] Heaton, D., Nichele, E., Clos, J., Fischer, J.E.: Perceptions of the agency and responsibility of the NHS COVID-19 app on Twitter: critical discourse analysis. J. Med. Internet Res. (2024). 10.2196/preprints.50388 10.2196/50388 PMC10836414 38300688

[117] Ferrara, E.: Should ChatGPT be biased? challenges and risks of bias in large language models. arXiv preprint arXiv:2304.03738 (2023)

[118] Lee, N.T., Resnick, P., Barton, G.: Algorithmic Bias Detection and Mitigation: Best Practices and Policies to Reduce Consumer Harms, vol. 2. Brookings Institute, Washington (2019)

[119] Donovan, J., Caplan, R., Matthews, J., Hanson, L.: Algorithmic accountability: a primer. Data & Society Research Institute (2018)

[120] Hartmann, J., Schwenzow, J., Witte, M.: The political ideology of conversational ai: Converging evidence on chatgpt’s pro-environmental, left-libertarian orientation. arXiv preprint arXiv:2301.01768 (2023)

[121] Whannel, K.: Could a chatbot answer prime minister’s questions? BBC (2022). https://www.bbc.co.uk/news/uk-politics-64053550

[122] Khalil, M., Er, E.: Will ChatGPT get you caught? rethinking of plagiarism detection. arXiv preprint arXiv:2302.04335 (2023)

[123] Lo, C.K.: What is the impact of ChatGPT on education? A rapid review of the literature. Educ. Sci. **13**(4), 410 (2023)

[124] Anders, B.A.: Is using chatgpt cheating, plagiarism, both, neither, or forward thinking? Patterns **4**, 3 (2023) 10.1016/j.patter.2023.100694 PMC10028419 36960444

[125] Heaton, D., Nichele, E., Clos, J., Fischer, J.E.: “The ChatGPT bot is causing panic now – but it’ll soon be as mundane a tool as Excel”: analysing topics, sentiment and emotions relating to ChatGPT on Twitter. Personal and Ubiquitous Computing (2024) (in press)

[126] Dai, Y., Liu, A., Lim, C.P.: Reconceptualizing ChatGPT and generative AI as a student-driven innovation in higher education. Procedia CIRP. **119**, 84–90 (2023)

[127] Roesslein, J.: Tweepy documentation. http://tweepy.readthedocs.io/en/v3 (2009)

[128] Kumar, S., Morstatter, F., Liu, H.: Twitter Data Analytics. Springer (2014)

[129] Jianqiang, Z.: Pre-processing boosting twitter sentiment analysis? IEEE Int. Conf. Smart City (2015). 10.1109/SmartCity.2015.158

[130] Chong, W.Y., Selvaretnam, B., Soon, L.: Natural language processing for sentiment analysis: an exploratory analysis on tweets. Int. Conf. Artif. Intell. Appl. Eng. Technol. (2014). 10.1109/icaiet.2014.43

[131] Woodfield, K., Morrell, G., Metzler, K., Blank, G., Salmons, J., Finnegan, J., Lucraft, M.: Blurring the boundaries? new social media, new social research: developing a network to explore the issues faced by researchers negotiating the new research landscape of online social media platforms. NCRM (2013)

[132] Jaworska, S.: Corpus approaches: investigating linguistic patterns and meanings. In: The Routledge Handbook of Language and Media, pp. 93–108. Routledge (2017)

[133] Baker, P.: Sociolinguistics and Corpus Linguistics. Edinburgh University Press (2010)

[134] Hunston, S.: How can a corpus be used to explore patterns? In: The Routledge Handbook of Corpus Linguistics, pp. 140–154. Routledge (2010)

[135] Anthony, L.: A critical look at software tools in corpus linguistics. Linguistic Res **30**(2), 141–161 (2013)

[136] Kopaczyk, J., Tyrkkö, J.: Applications of Pattern-driven Methods in Corpus Linguistics, vol. 82. John Benjamins Publishing Company (2018)

[137] Nugraha, I.S., Sujatna, E.T.S., Mahdi, S.: Corpus linguistic study of tweets using #charliehebdo hashtag. J. Appl. Linguist. Lit. **5**(1), 54–70 (2021)

[138] Kopf, S., Nichele, E.: Es-tu charlie? Doing Polit. **80**, 211 (2018)

[139] Russo, K.E., Grasso, A.: Coping with dis/ableism in twitter discourse: a corpus-based critical appraisal analysis of the hidden disabilities sunflower lanyard case. Int. J. Lang. Stud. **16**, 4 (2022)

[140] Mautner, G.: Mining large corpora for social information: the case of elderly. Lang. Soc. **36**(1), 51–72 (2007)

[141] Hoey, M.: Grammatical creativity: A corpus perspective. M. Hoey et al.(eds.), 31–56 (2007)

[142] Kilgarriff, A., Rychlý, P., Smrž, P., Tugwell, D.: Itri-04-08 the sketch engine. Information Technology (2004)

[143] Johnson, M.N.P., McLean, E., Kobayashi, A.: Discourse analysis. In: International Encyclopedia of Human Geography, 2nd edn., 377–383. Elsevier, Oxford (2020). 10.1016/b978-0-08-102295-5.10814-5

[144] Hart, C.: Critical discourse analysis and metaphor: toward a theoretical framework. Crit. Discourse Stud. **5**(2), 91–106 (2008)

[145] Kendall, G., et al.: What is critical discourse analysis? In: Forum Qualitative Sozialforschung/Forum: Qualitative Social Research, vol. 8 (2007)

[146] Van Dijk, T.A.: What is political discourse analysis. Belgian J. Linguist. **11**(1), 11–52 (1997)

[147] Tenorio, E.H.: Critical discourse analysis, an overview. Nordic J. Engl. Stud. **10**(1), 183–210 (2011)

[148] Bloor, M., Bloor, T.: The Practice of Critical Discourse Analysis: An Introduction. Routledge (2013)

[149] Aljarallah, R.S.: A critical discourse analysis of twitter posts on the perspectives of women driving in saudi arabia. Technical report, Arizona State University (2017)

[150] Sveinson, K., Allison, R.: Something seriously wrong with us soccer: a critical discourse analysis of consumers twitter responses to us soccers girls apparel promotion. J. Sport Manag. **1**, 1–13 (2021)

[151] Kreis, R.: # refugeesnotwelcome: anti-refugee discourse on twitter. Discourse Commun. **11**(5), 498–514 (2017)

[152] Baker, P.: Acceptable bias? Using corpus linguistics methods with critical discourse analysis. Crit. Discourse Stud. **9**(3), 247–256 (2012)

[153] Nartey, M., Mwinlaaru, I.N.: Towards a decade of synergising corpus linguistics and critical discourse analysis: a meta-analysis. Corpora **14**(2), 203–235 (2019)

[154] Abbas, A., Zahra, T.: Corpus driven critical discourse analysis of 2020 presidential election campaign tweets of donald trump and joe biden. Hayatian J. Linguist. Lit. **5**(1), 26–47 (2021)

[155] Weber, M.: Max Weber: Selections in Translation. Cambridge University Press (1978)

[156] McGlashan, M.: Collective identity and discourse practice in the followership of the football lads alliance on Twitter. Discourse Soc. **31**(3), 307–328 (2020)

[157] Fadanelli, S.B., Dal Pozzo, D.F., Fin, C.C.: The representation of social actors in the tweets of Jair Messias Bolsonaro. Antares **12**, 74–99 (2020)

[158] Bernard, T.: The discursive representation of social actors in the corporate social responsibility (CSR) and integrated annual (IA) reports of two South African mining companies. Crit. Approaches Discourse Anal. Across Discip. **10**, 1 (2018)

[159] Razis, G., Anagnostopoulos, I., Saloun, P.: Thematic labeling of Twitter accounts using dbpedia properties. In: 2016 11th International Workshop on Semantic and Social Media Adaptation and Personalization (SMAP), IEEE, pp. 106–111 (2016)

[160] Kitishat, A.R., Al Kayed, M., Al-Ajalein, M.: A corpus-assisted critical discourse analysis of the Syrian refugee crisis in Jordanian newspapers. Int. J. Engl. Linguist. **10**, 6 (2020)

[161] Van Dijk, T.A.: Discourse, ideology and context. Folia Linguistica **35**(1–2), 11–40 (2001)

[162] Goatly, A.: Washing the Brain: Metaphor and Hidden Ideology, vol. 23. John Benjamins Publishing (2007)

[163] Coeckelbergh, M.: AI Ethics. MIT Press (2020)

[164] Dwivedi, Y.K., Hughes, L., Ismagilova, E., Aarts, G., Coombs, C., Crick, T., Duan, Y., Dwivedi, R., Edwards, J., Eirug, A., et al.: Artificial intelligence (AI): Multidisciplinary perspectives on emerging challenges, opportunities, and agenda for research, practice and policy. Int. J. Inf. Manag. **57**, 101994 (2021)

[165] Laitinen, A., Sahlgren, O.: AI systems and respect for human autonomy. Front. Artif. Intell. **4**, 151 (2021) 10.3389/frai.2021.705164 PMC8576577 34765969

[166] Wetherell, M., Potter, J.: Discourse analysis and the identification of interpretative repertoires. In C. Antaki (ed.) Analysing everyday explanation: a casebook of methods, pp. 168–183. Sage, Newbury Park (1988)

[167] Gill, R.: Discourse analysis. Qual. Res. Text Image Sound **1**, 172–190 (2000)

[168] Morgan, A.: Discourse analysis: an overview for the neophyte researcher. J. Health Soc. Care Improv. **1**(1), 1–7 (2010)

